# Bioinspired enzymatic polyphenolic nanoplatform for redox modulation and functional renal restoration in diabetic nephropathy

**DOI:** 10.1093/rb/rbag102

**Published:** 2026-05-26

**Authors:** Linli Cai, Tianyou Wang, Yin Huang, Dehong Cao, Fenghao Yang, Xingyuan Li, Yutong Zou, Qing Yang, Maoyun Li, Zhipeng Gu, Fang Liu

**Affiliations:** Department of Nephrology, West China Hospital of Sichuan University, Chengdu 610041, China; Laboratory of Diabetic Kidney Disease, Kidney Research Institute, Department of Nephrology, West China Hospital of Sichuan University, Chengdu 610093, China; College of Polymer Science and Engineering, State Key Laboratory of Advanced Polymer Materials, Sichuan University, Chengdu 610065, China; Department of Urology, West China Hospital of Sichuan University, Chengdu 610041, China; Department of Urology, West China Hospital of Sichuan University, Chengdu 610041, China; Department of Clinical Medicine, Southwest Medical University, Luzhou 646000, China; Department of Nephrology, West China Hospital of Sichuan University, Chengdu 610041, China; Laboratory of Diabetic Kidney Disease, Kidney Research Institute, Department of Nephrology, West China Hospital of Sichuan University, Chengdu 610093, China; Department of Nephrology, West China Hospital of Sichuan University, Chengdu 610041, China; Laboratory of Diabetic Kidney Disease, Kidney Research Institute, Department of Nephrology, West China Hospital of Sichuan University, Chengdu 610093, China; Department of Nephrology, West China Hospital of Sichuan University, Chengdu 610041, China; Huaxi MR Research Center, Department of Radiology, Frontiers Science Center for Disease-Related Molecular Network, State Key Laboratory of Biotherapy, West China Hospital, Sichuan University, Chengdu 610041, China; Laboratory of Diabetic Kidney Disease, Kidney Research Institute, Department of Nephrology, West China Hospital of Sichuan University, Chengdu 610093, China; Department of Nephrology, West China Hospital of Sichuan University, Chengdu 610041, China; Laboratory of Diabetic Kidney Disease, Kidney Research Institute, Department of Nephrology, West China Hospital of Sichuan University, Chengdu 610093, China

**Keywords:** polyphenolic nanomaterials, enzymatic polymerization, active molecule delivery, oxidative stress regulation, diabetic nephropathy

## Abstract

Oxidative stress is a central pathological driver in diabetic nephropathy (DN), leading to excessive reactive oxygen species (ROS) generation, chronic inflammation and progressive renal fibrosis. Effective modulation of oxidative imbalance thus represents a vital therapeutic strategy for mitigating DN progression. Here, we developed a bioinspired polyphenolic nanoplatform by first constructing polymerized grape seed polyphenol nanoparticles via enzymatic polymerization, followed by supramolecular assembly with the bioactive polyphenol resveratrol. This design combined the intrinsic bioactivity of polyphenols with active resveratrol integration, yielding a stable and biocompatible strategy that alleviated DN and enabled superior restoration of renal structure and function. In diabetic models, it promoted coordinated recovery of renal morphology, key structural proteins and physiological function, suggesting potential advantages for long-term renal protection beyond short-term biochemical improvement. These findings demonstrated that enzymatically engineered polyphenolic nanotherapeutics provided a potent and biocompatible approach for oxidative regulation and functional renal restoration in DN therapy.

## Graphical abstract

**Graphical Abstract:**
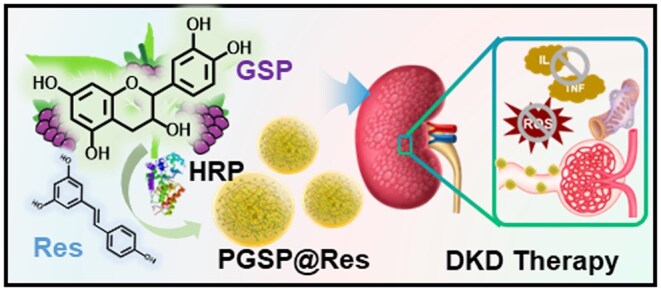

## Introduction

Diabetic nephropathy (DN), one of the most severe microvascular complications of diabetes mellitus, represents the leading cause of end-stage renal disease worldwide [1, 2]. The pathogenesis of DN is multifactorial, involving hyperglycemia-induced metabolic dysregulation, oxidative stress and chronic inflammation that collectively damage glomerular and tubular structures [3–7]. In the early stages, glomerular hypertrophy, mesangial expansion and thickening of the basement membrane occur, followed by progressive fibrosis, tubular atrophy and renal functional decline [8–11]. Clinically, DN manifests as persistent proteinuria, elevated serum creatinine (CREA) and eventual renal failure. Despite the availability of hypoglycemic and antihypertensive agents, conventional therapies such as insulin, angiotensin-converting enzyme (ACE) inhibitors or SGLT2 inhibitors primarily alleviate systemic metabolic stress [12–15]. Consequently, the incidence of DN continues to rise, underscoring the urgent need for novel therapeutic strategies that can directly intervene in microenvironment balance and renal tissue injury.

Accumulating evidence indicates that oxidative stress acts as a pivotal pathological trigger and amplifier in DN [7, 16, 17]. Persistent hyperglycemia enhances mitochondrial electron leakage and activates nicotinamide adenine dinucleotide phosphate (NADPH) oxidase, leading to excessive production of reactive oxygen species (ROS). These radicals overwhelm the endogenous antioxidative defense systems, such as superoxide dismutase (SOD) and catalase (CAT), resulting in lipid peroxidation, protein oxidation and DNA damage [18–20]. The resultant oxidative injury further promotes inflammatory cytokine release and apoptosis of renal podocytes as well as tubular epithelial cells, establishing a vicious cycle that accelerates glomerulosclerosis and tubulointerstitial fibrosis [21–25]. Therefore, reestablishing redox homeostasis and suppressing ROS-driven inflammation have emerged as promising approaches for halting DN progression.

Natural polyphenols, a broad class of secondary metabolites abundant in plants such as tea, grapes and berries, exhibit potent antioxidative and anti-inflammatory activities through direct radical scavenging and signaling pathway modulation [26–32]. The multiple phenolic hydroxyl groups enable these molecules to donate electrons or hydrogen atoms to neutralize ROS [26, 33]. Beyond their inherent biochemical activity, polyphenols have gained attention as versatile building blocks for functional biomaterials. Their strong adhesion, coordination and self-polymerization properties have been exploited to design bioinspired coatings, hydrogels and nanoplatforms for biomedical applications including wound dressings, photothermal therapy and controlled drug release [34–40]. In addition to the broad therapeutic effects of polyphenols, the introduction of specific polyphenolic blocks with well-established biofunctions has been shown to further refine pathological indicators, offering enhanced physiological support for DN related disease improvement [41, 42]. However, the inherent instability, easy oxidation and poor solubility of natural polyphenols restrict their direct biomedical translation, particularly under physiological conditions where their antioxidative capacity rapidly diminishes [29, 42, 43]. Recent advances in enzymatic polymerization offer an elegant and eco-friendly approach to overcome these drawbacks. Horseradish peroxidase (HRP)-mediated coupling reactions can catalyze phenolic monomers into crosslinked polymeric networks under mild conditions, enhancing the chemical stability and bioactivity of polyphenol-based materials [44]. The resulting polyphenol nanoparticles (NPs) not only maintain intrinsic functionality but also provide abundant surface sites for secondary modifications or therapeutic molecule encapsulation, making them promising candidates for targeted antioxidative therapy [45, 46].

In this work, a multifunctional polyphenolic nanoplatform was developed via HRP-catalyzed enzymatic polymerization of grape seed polyphenols (GSP), followed by loading with hydrophobic polyphenolic resveratrol (Res), which could mitigate nephropathy by activating signaling, promoting autophagy, alleviating endoplasmic reticulum stress, improving lipid metabolic dysregulation and preserving key glomerular and podocyte structural proteins essential for renal function [41, 47]. The enzymatic polymerization process enabled the formation of stable PGSP NPs with preserved antioxidative moieties, while the incorporation of resveratrol for PGSP@Res construction enhanced the overall therapeutic potency through synergistic physiological microenvironment modulation. The obtained PGSP@Res NPs exhibited uniform morphology, high colloidal stability and efficient radical scavenging ability. More importantly, through combination of *in vitro* and *in vivo* studies, it demonstrated that PGSP@Res could effectively mitigate oxidative damage, suppress inflammation and inhibit apoptosis in renal cells and tissues of diabetic mice. Mechanistically, the nanoplatform promoted endogenous antioxidative enzyme expression and restored renal oxidative balance, ultimately preserving glomerular structure and renal function. This study not only presents a sustainable and bioinspired method to engineer polyphenolic nanotherapeutics via enzymatic polymerization and polyphenol assembly, but also introduces a distinctive strategy that integrates general and specific polyphenol bioactivities within a single platform. Such a design enables simultaneous modulation of pathological processes and coordinated restoration of renal structure and function, thereby providing new insights into therapeutic strategies for DN and other complex diseases.

## Experimental section

### Materials

GSP (98%) was purchased from DASF Bio-Technology Co., Ltd (Nanjing, China). HRP (>300 U/mg) and potassium persulfate (99%) were obtained from Shanghai Aladdin Bio-Chem Technology Co., Ltd (Shanghai, China). Hydrogen peroxide (30%) and sodium chloride (99%) were supplied by Chengdu Jinshan Chemical Reagent Co., Ltd (Chengdu, China). 2,2-Diphenyl-1-picrylhydrazyl (DPPH, 95%) was obtained from Alfa Aesar (Shanghai, China), and 2,2′-azino-bis(3-ethylbenzothiazoline-6-sulfonic acid) diammonium salt (ABTS, 98%) was purchased from TCI (Tokyo, Japan). Ethanol (analytical grade) was purchased from Titan Technology Co., Ltd (Shanghai, China). All chemicals were of analytical grade and used as received without further purification. Deionized water was used throughout all experiments.

### Preparation of PGSP and PGSP@Res NPs

PGSP NPs were synthesized through an HRP-catalyzed oxidative self-polymerization of GSP [44]. Briefly, 450 mg of GSP was completely dissolved in 127.5 mL of deionized water, while 12.9 mg of HRP was dispersed in 22.5 mL of ethanol to form a homogeneous catalytic solution. Under gentle stirring, 4.9 mL of 0.3% H_2_O_2_ solution was added dropwise to the mixed system. A rapid color transition from deep red to dark brown occurred within seconds, indicating the onset of enzymatic oxidation and polymerization. The reaction was maintained at 65°C under constant stirring for 6 h. The resulting PGSP nanoparticles were collected by centrifugation (15 000 rpm, 10 min) and sequentially washed 3 times with saturated saline and deionized water to remove unreacted monomers, salts and residual enzymes, ensuring product purity and stability. For Res loading, a 10-mg/mL Res solution in DMSO was prepared and added dropwise into the aqueous dispersion of PGSP NPs under ultrasonication. Different Res-to-PGSP feeding ratios were screened to optimize loading capacity and encapsulation efficiency. The mixture was stirred for 12 h at room temperature and then centrifuged (15 000 rpm, 10 min) followed by three washing cycles with deionized water. The final product was re-dispersed in water and stored at 4°C for further use.

### Cell culture

Human proximal tubular epithelial cells (HK-2) were used for all *in vitro* experiments. The cells were cultured in HK-2-specific complete medium (Procell) under standard conditions of 37°C, 5% CO_2_ and saturated humidity. All procedures were conducted under sterile conditions. After thawing, cell attachment and growth status were monitored within 24 h to evaluate recovery efficiency. The medium was replaced every 2–3 days depending on cell density and nutrient consumption, and cells were passaged at 80–90% confluence.

### Live/dead cell staining

After treatments, the culture medium was removed, and cells grown on glass coverslips were washed 1–3 times with phosphate-buffered saline (PBS, pH 7.4, Solarbio). A Calcein AM/PI working solution (Beyotime, China) was added to cover the cells, followed by incubation at 37°C in the dark for 30 min. After incubation, cells were washed 3 times with PBS on a shaker for 5 min each. Coverslips were mounted using an antifade reagent and observed under a fluorescence microscope (Nikon Eclipse C1). Live cells stained with Calcein AM exhibited green fluorescence (Ex/Em = 494/517 nm), whereas dead cells stained with PI displayed red fluorescence (Ex/Em = 535/617 nm).

### Flow cytometry analysis of apoptosis

Apoptosis was quantified using an Annexin V-FITC/PI dual-staining kit (eBioscience). HK-2 cells were harvested, resuspended in binding buffer at a concentration of 1 × 10^6^ cells mL^−1^, and centrifuged at 300 rpm for 5 min. The cell pellet was resuspended in 100 μL of fresh binding buffer, followed by the addition of 5 μL Annexin V-FITC and incubation for 10 min at room temperature in the dark. Subsequently, 10 μL PI solution was added and incubated for another 5 min in the dark. After adding 400 μL PBS, samples were immediately analyzed by flow cytometry. Data were processed using FlowJo software to determine apoptotic ratios.

### Quantitative analysis of oxidative Stress-Related gene expression

Total RNA was extracted from HK-2 cells using the Trizol method (RNAiso Plus, Takara) following the manufacturer’s protocol. Briefly, cells were lysed in 1 mL Trizol reagent and incubated for 5 min at room temperature, followed by the addition of 0.2 mL chloroform. After vigorous shaking and phase separation by centrifugation (12 000 rpm, 10 min, 4°C), the aqueous phase was transferred to a new microtube, and RNA was precipitated with 0.5 mL isopropanol. The RNA pellet was washed with 75% pre-cooled ethanol, air-dried for 5–10 min and dissolved in 30–50 μL RNase-free water. RNA concentration and purity were determined using a NanoDrop 2000 spectrophotometer (Thermo Fisher Scientific). Quantitative real-time PCR (qRT-PCR) was performed using SYBR^®^ Select Master Mix (Applied Biosystems) according to the manufacturer’s instructions. Primer sequences were listed in Supplementary Table S1 (including *SOD2, CAT, Hmox1, IL-6, TNF-α*).

### Ethical statement

All animal experiments were conducted in accordance with the guidelines and regulations approved by the ethics committee of West China Hospital, Sichuan University (Approval number: 20260121008). Male C57BL/6J mice (8 weeks old) were used for acute toxicity assessment, while male db/db mice (BKS.Cg-+Leprdb/+Leprdb/J) aged 8 weeks served as a model for type 2 diabetes mellitus. Age-matched male db/m mice were used as wild-type (WT) controls. C57BL/6J mice were purchased from Beijing HFK Bioscience Co., Ltd, and db/db mice were obtained from Cyagen Biosciences Inc. All animals were maintained in the SPF-grade animal facility under controlled temperature (22 ± 2°C), humidity (50 ± 10%) and a 12-h light/dark cycle, with free access to standard rodent chow and sterile drinking water.

### Therapeutic evaluation towards DN

For efficacy assessment, all db/db mice were maintained until 10 weeks of age to ensure stable hyperglycemia prior to treatment. The mice were then randomly divided into three groups (*n* = 3 per group): untreated diabetic control (Model), PGSP (5 mg/kg) and PGSP@Res (5 mg/kg). Treatments were administered via tail vein injection. For acute pharmacodynamics evaluation, samples were collected 24 h after the first injection (Day 1). For short-term efficacy, injections were performed on Days 3 and 6 and samples were collected on Day 7. For long-term efficacy, injections were administered on Days 9, 12 and 15, with terminal collection on Day 18. At each time point, body weight and blood glucose were recorded, and samples including blood, urine, heart, liver, kidney and pancreas were collected for analysis.

### Histopathological staining

For Hematoxylin and eosin (H&E) staining, paraformaldehyde-fixed tissues were dehydrated, paraffin-embedded and sectioned. Sections were deparaffinized, rehydrated, stained with hematoxylin (5–10 min), differentiated with acid alcohol, blued in weak alkaline solution, counterstained with eosin (3 min), dehydrated through graded alcohols, cleared in xylene and mounted with neutral resin. For PAS staining, deparaffinized sections were oxidized in periodic acid for 5–10 min, stained with Schiff’s reagent for 10–20 min, washed under running water for 10 min, counterstained with hematoxylin (1–3 min), differentiated, blued and mounted. For Masson’s trichrome staining, sections were treated with potassium dichromate overnight, incubated at 63°C for 1 h, stained with Ponceau acid fuchsin for 10 min, briefly rinsed, differentiated with phosphomolybdic acid (a few seconds to 2 min), stained with aniline blue for 2 min, dehydrated, cleared and mounted.

### Biochemical assays of renal oxidative stress (SOD, MDA, GSH, CAT, CREA)

Tissue homogenates were prepared by mixing tissues with saline at a 1:9 (w/v) ratio. Homogenization was performed mechanically at 4000 rpm for 10 min, followed by centrifugation. The supernatant (10% homogenate) was used for biochemical analysis. SOD, malondialdehyde (MDA), CAT, reduced glutathione (GSH) and CREA levels were determined using commercial assay kits (Nanjing Jiancheng Bioengineering Institute, China) according to the manufacturers’ protocols.

### Serum and urine ELISA assays

Serum and urine levels of glycated serum protein (GSP), interleukin-6 (IL-6), tumor necrosis factor-α (TNF-α), kidney injury molecule-1 (Kim-1), neutrophil gelatinase-associated lipocalin (NGAL) and microalbumin/albumin ratio (MAU/ALB) were measured by ELISA. Briefly, all reagents and samples were equilibrated to room temperature for 30 min. Standard and sample wells were set accordingly. Fifty microliters of standards or 10 μL of samples (diluted 1:5 with sample diluent) were added per well, followed by 100 μL HRP-conjugated detection antibody. Plates were sealed and incubated at 37°C for 60 min, washed 5 times and then incubated with substrates A and B (50 μL each) for 15 min at 37°C in the dark. The reaction was stopped with 50 μL stop solution, and absorbance was read at 450 nm within 10 min using a microplate reader. All ELISA kits were obtained from ZCIBIO Technology Co., Ltd.

### Renal tissue ROS detection

Frozen kidney sections were equilibrated to room temperature and air-dried. Autofluorescence was quenched for 5 min and rinsed with running water for 10 min. The sections were then incubated with ROS staining solution for 30 min in the dark, washed 3 times with PBS and mounted using an antifade reagent for fluorescence microscopy imaging.

### Western blot analysis of renal proteins

Kidney tissues were washed with pre-cooled PBS to remove blood residues and minced on ice. Samples were homogenized with lysis buffer containing protease inhibitors and PMSF using a bead mill (2–5 min). Lysates were incubated on ice for 30 min with occasional mixing, then centrifuged at 12 000 rpm for 10 min at 4°C. Supernatants were collected for protein quantification using a BCA assay (Biosharp). Equal amounts of protein were denatured with 5× loading buffer at 95°C for 10 min and stored at −80°C. Proteins were separated by SDS-PAGE and transferred onto PVDF membranes. Membranes were blocked, incubated overnight at 4°C with primary antibodies, washed and incubated with HRP-conjugated secondary antibodies for 2 h at room temperature. Bands were visualized using a Tanon chemiluminescence imaging system, and relative intensities were quantified using Gel-Pro Analyzer software. Primary antibodies included: Bax (A19684, Abclonal), Bcl-2 (ab182858, Abcam), Collagen IV (A24008, Abclonal), Nephrin (HA722886, Huabio), Podocin (ET7107-34, Huabio), α-smooth muscle actin (α-SMA) (GB111364-50, Servicebio) and β-actin (AC026, Abclonal).

### Statistical analysis

Student *t*-tests and one-way ANOVA tests were performed and statistical plots were drawn using GraphPad Prism 9 and Origin. The significance of multiple comparisons was tested using Tukey’s or Dunnett’s multiple comparison tests. And *P-*value <0.05 was considered a statistically significant difference.

## Results and discussion

### Design and characterization of polyphenolic nanoplatform

**Figure 1:**
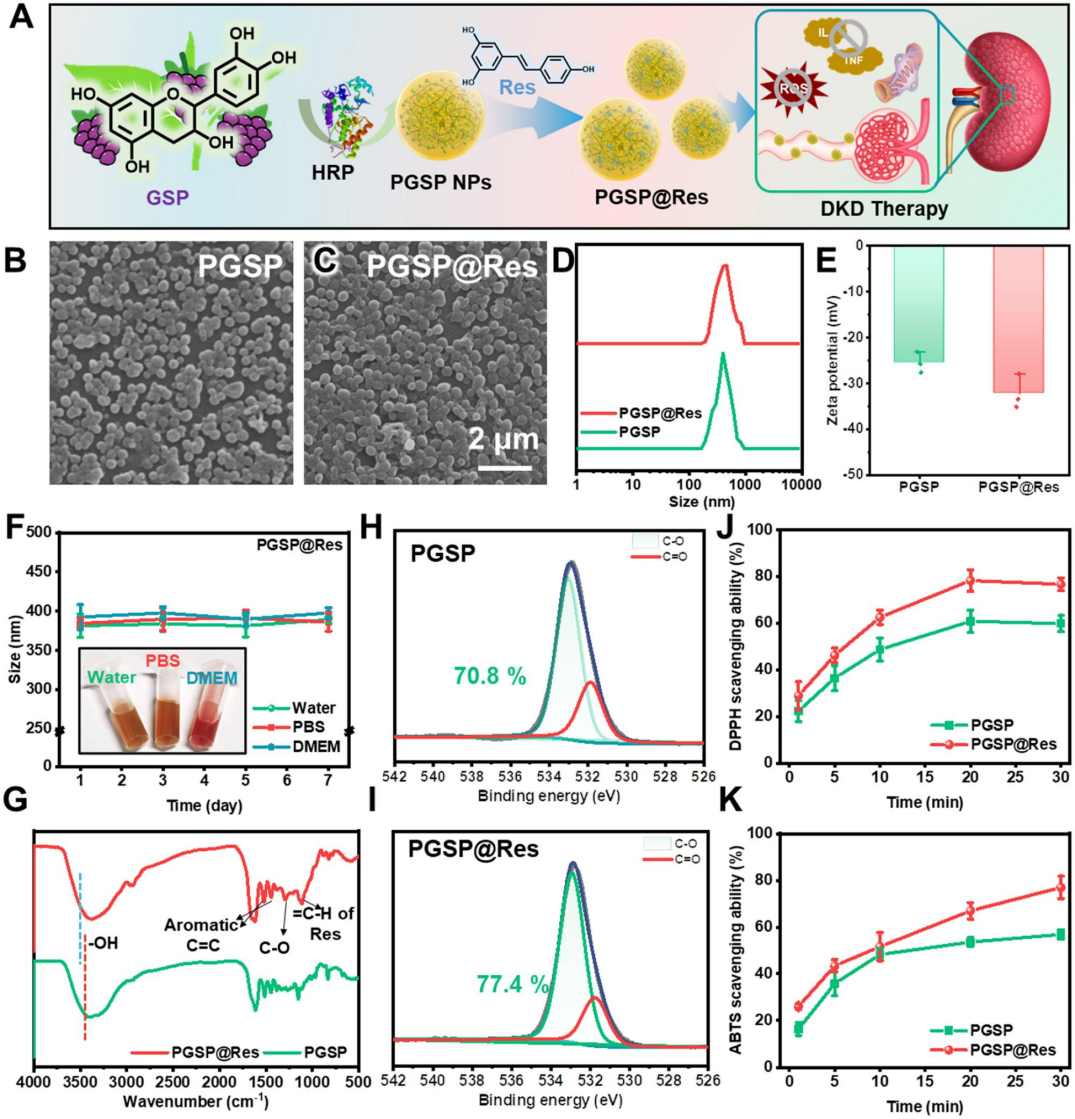
Design and characterization of PGSP@Res NPs. (**A**) Schematic illustration of the fabrication process and therapeutic concept. (**B, C**) SEM images, (**D**) DLS analysis and (**E**) Zeta potential analysis of PGSP and PGSP@Res NPs. (**F**) Size stability of PGSP@Res NPs in water, PBS and DMEM over 7 days, and the corresponding optical images. (**G**) FTIR spectra and (**H, I**) XPS O 1s spectra of PGSP and PGSP@Res NPs. (**J, K**) Time-dependent DPPH and ABTS radical-scavenging assays over 30 min of PGSP and PGSP@Res NPs.

Inspired by the intrinsic antioxidative and bioactive properties of natural polyphenols, a multifunctional nanoplatform for DN therapy was constructed based on enzymatically GSP polymerization. As illustrated in Figure 1A, GSP was catalytically polymerized by HRP under mild conditions, yielding uniform PGSP NPs. Under HRP catalysis, phenolic hydroxyl groups are oxidized to phenoxy radicals, which subsequently undergo coupling reactions to form oligomeric polyphenol structures. As previously reported, PGSP NPs are mainly composed of such oligomeric units and representative structures are shown in Supplementary Figure S1 [44]. To further enhance therapeutic efficacy, the bioactive yet hydrophobic compound Res was loaded onto the NPs through hydrophobic and hydrogen-bond interactions, forming PGSP@Res NPs (Supplementary Figure S2). The release study demonstrated that Res could be gradually released under physiological conditions, which could be attributed to the dynamic non-covalent interactions within the polyphenol network, enabling sustained release behavior (Supplementary Figure S3). This biomimetic strategy integrated the intrinsic ROS scavenging capacity of polyphenols with the pathway regulating potential of Res, offering a synergistic avenue for DN treatment.

The morphological features of PGSP and PGSP@Res NPs were first investigated by scanning electron microscopy (SEM). Both NPs displayed homogeneous and well-dispersed spherical morphologies with diameters around 250 nm (Figure 1B and C). The absence of aggregation or structural collapse after Res encapsulation indicated that the enzymatically polymerized framework provides robust structural integrity. This observation was corroborated by dynamic light scattering (DLS) results (Figure 1D), which showed negligible variation in hydrodynamic size distribution between PGSP and PGSP@Res, confirming that Res loading did not alter the dispersibility and uniformity. Surface charge analysis revealed that both PGSP and PGSP@Res possessed negative zeta potentials (Figure 1E), a characteristic attributed to the abundant phenolic hydroxyl on the NPs surface. Consistently, the stability tests demonstrated negligible size fluctuations over 7 days in different physiological environments (Figure 1F and Supplementary Figure S4), implying excellent colloidal stability under physiological conditions. Notably, optical images further confirmed the absence of visible aggregation or precipitation, with the dispersions maintaining a uniform and stable state over time. Importantly, the incorporation of hydrophobic Res did not significantly compromise the aqueous dispersibility of the system, as PGSP@Res remained well-dispersed not only in water but also in physiologically relevant media without observable aggregation, which could be attributed to the preserved hydrophilic phenolic functionalities within the polyphenol network.

Fourier transform infrared (FTIR) spectra further elucidated the intermolecular interactions between the NPs and Res (Figure 1G). The broad O–H stretching at around ∼3200–3500 cm^−1^ exhibited a slight red shift accompanied by attenuation in the phenolic vibration region, implying the formation of hydrogen bonds between Res and the phenolic components of PGSP NPs [48]. The preserved aromatic C=C stretching peaks at ∼1600 and ∼1510 cm^−1^ and C–O vibrations at ∼1150cm^−1^ suggest that the polyphenolic framework remained intact. Meanwhile, subtle spectral changes, including weak features (rans-olefinic =C–H bending of Res at ∼960 cm^−1^) attributable to Res, further support its successful integration via non-covalent interactions including hydrogen bonding and π-π stacking. This interaction not only favored encapsulation but also enhanced the interfacial compatibility. X-ray photoelectron spectroscopy (XPS) analyses provided additional evidence for successful Res loading. The O 1s spectra of PGSP and PGSP@Res (Figure 1H and I and Supplementary Figure S5) showed a marked increase in the C–O signal proportion after encapsulation, indicating the enrichment of phenolic hydroxyl group on the NPs surface.

Given that oxidative stress is a major pathological driver in DN, the antioxidative potential of the NPs was evaluated using DPPH and ABTS radical scavenging assays [44, 49]. As shown in Figure 1J and K, both PGSP and PGSP@Res exhibited time-dependent radical scavenging activities, while PGSP@Res consistently demonstrated higher efficiencies in both assays. The radical scavenging activity of PGSP@Res was governed by hydrogen atom/electron transfer from phenolic hydroxyl groups, forming stabilized phenoxyl radicals and partial quinone structures [26]. The incorporation of Res increased the density of active sites and enhanced π-conjugation, thereby improving radical stabilization and antioxidant performance. These results suggested that Res loading could reinforce the antioxidative capability of the nanoplatform, thereby enhancing its potential to alleviate oxidative stress-related renal injury.

### *In vitro* cytoprotective effects against oxidative stress and physiological toxicity

Following the successful fabrication and characterization of PGSP@Res NPs, their biocompatibility and cellular protective effects under oxidative stress were next assessed, which are essential prerequisites for therapeutic application. HK-2, a commonly used *in vitro* model for DN studies, were employed due to their relevance in reflecting tubular injury, oxidative stress and inflammatory responses under pathological conditions. As shown in Supplementary Figure S6, the cytotoxicity of GSP, Res, PGSP and PGSP@Res was systematically evaluated across a wide concentration range after 48 h. Free Res exhibited significant dose-dependent cytotoxicity, with a sharp reduction in cell viability observed even at 25 µg/mL, likely due to its limited solubility and membrane perturbation effects. In contrast, GSP and PGSP maintained excellent cytocompatibility over the tested range, while PGSP@Res showed markedly reduced toxicity compared to free Res. Flow cytometry and live/dead staining results (Supplementary Figure S7) corroborated these findings, demonstrating consistent trends in cellular viability.

**Figure 2:**
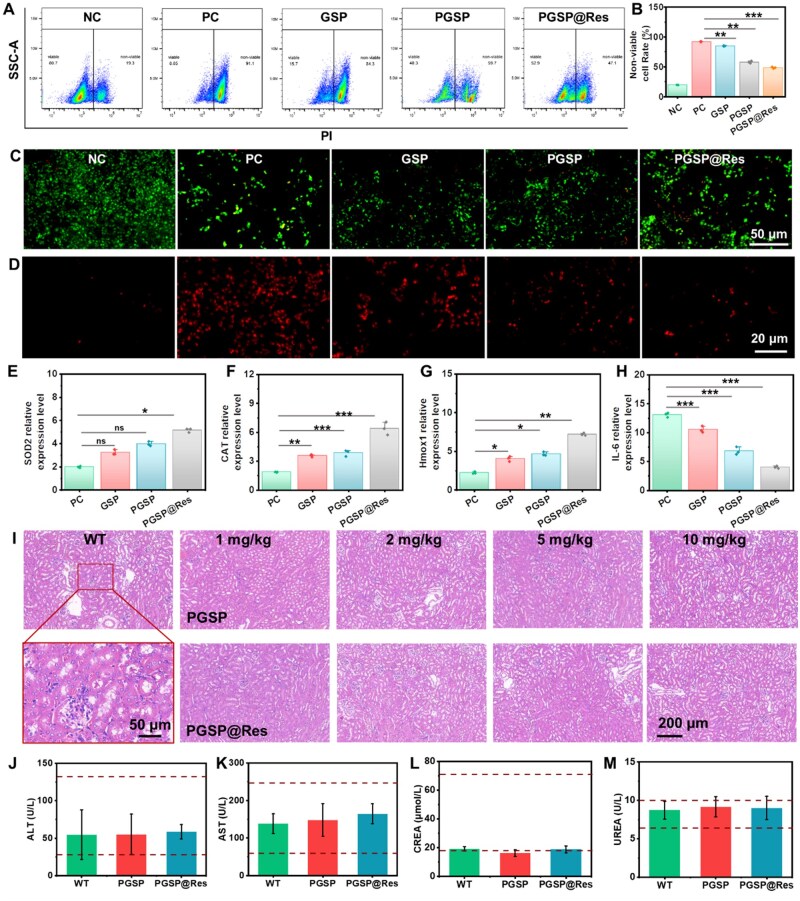
*In vitro* cytoprotective performance and *in vivo* biosafety of PGSP@Res NPs. (**A**) Flow cytometry analysis of hydrogen peroxide induced oxidative stress cells after different treatments. (**B**) Quantification of non-viable cells derived from flow cytometry analysis. (**C**) Live/dead fluorescence staining images across various treatment groups. (**D**) Intracellular ROS fluorescence images. Relative mRNA expression of antioxidant-related enzymes (**E**) SOD2, (**F**) CAT, (**G**) Hmox1 and (**H**) proinflammatory cytokine IL-6 (*n* = 3). (**I**) H&E staining of kidney tissues after administration of PGSP or PGSP@Res at various doses (1–10 mg/kg). (**J–M**) Serum biochemical analyses of ALT, AST, CREA and UREA for systemic biocompatibility evaluation. The ns represents no significant difference; *P < 0.05; **P < 0.01; ***P < 0.001.

To simulate oxidative stress conditions relevant to DN, an optimized hydrogen peroxide induced stress model was first established (Supplementary Figure S8). Subsequent co-incubation of stressed cells with different materials revealed distinct cytoprotective trends. Cell viability assays (Supplementary Figure S9) and quantitative flow cytometry (Figure 2A and B) confirmed that all polyphenol-containing groups alleviated oxidative damage compared with the positive control (PC) group, with PGSP@Res demonstrating the most pronounced protective effect. The live/dead fluorescence images (Figure 2C) further visualized this trend, showing more cells and fewer propidium iodide (PI)-positive (dead) cells in the PGSP@Res-treated group, consistent with its enhanced radical scavenging capability observed in Figure 1J and K. Intracellular ROS staining images (Figure 2D) provided direct evidence of oxidative modulation. Cells treated with PGSP@Res displayed significantly reduced red fluorescence intensity, indicating efficient ROS suppression. The synergistic antioxidative activity likely aroused from the dual functionality of the polyphenol substrate and the entrapped Res, enabling continuous radical quenching.

To elucidate the molecular mechanisms underlying the observed protective effects, oxidative stress related genes were analyzed by quantitative real-time polymerase chain reaction (qRT-PCR). The expression levels of primary enzymatic antioxidants of SOD2 responsible for superoxide and CAT responsible for hydrogen peroxide were markedly upregulated following PGSP@Res treatment (Figure 2E and F). The enhancement indicated that PGSP@Res not only provided direct radical scavenging but also activated endogenous antioxidative defense systems. In addition, the expression of heme oxygenase 1 (Hmox1), a downstream effector of the Nrf2 signaling pathway, was significantly elevated (Figure 2G), suggesting activation of the cellular oxidative regulatory cascade. Conversely, the proinflammatory cytokine IL-6, a critical mediator of renal inflammation in DN, was strongly downregulated (Figure 2H). These results collectively demonstrated that PGSP@Res NPs could mitigate oxidative injury and inflammatory responses through both direct ROS neutralization and indirect transcriptional regulation of antioxidative and anti-inflammatory pathways.

Before proceeding to therapeutic experiments in DN models, *in vivo* biosafety was comprehensively evaluated. H&E staining of kidney sections (Figure 2I) revealed normal glomerular and tubular structures across treatment groups and dosages, with no observable pathological lesions at 5 mg/kg. In contrast, when the dose was increased to 10 mg/kg, partial detachment of the tubular brush border was observed in renal tissue sections, suggesting potential dose-dependent structural perturbation. Although the reversibility of this alteration was not specifically investigated, it indicated that higher dosages might induce transient renal stress. Importantly, at 5 mg/kg, no histopathological abnormalities were detected throughout the 18-day treatment period, further confirming its biosafety. Based on these findings, 5 mg/kg was selected as the optimal dose for subsequent therapeutic studies. Moreover, histological examinations of major organs (including heart, liver, spleen and lung, Supplementary Figure S10) showed no structural abnormalities, further confirming systemic safety. On the other hand, serum biochemical analyses demonstrated that liver function markers including alanine transaminase (ALT) and aspartate aminotransferase (AST) as well as renal function indicators including CREA and UREA remained within physiological ranges following administration of both PGSP and PGSP@Res, comparable to WT controls (Figure 2J–M). These findings confirmed that the enzymatically polymerized polyphenolic nanomaterials possessed excellent *in vivo* compatibility and negligible toxicity and provided a strong basis for the subsequent therapeutic assessment in DN models.

### *In vivo* therapeutic efficacy of PGSP@Res NPs in DN

Encouraged by the excellent antioxidative and cytoprotective effects of PGSP@Res *in vitro*, the therapeutic performance was next evaluated in a DN mouse model. The male db/db (BKS.Cg-+Leprdb/+Leprdb/J) mice were used as DN models (Model group), while age-matched db/m mice served as WT controls (WT group). After successful model establishment, mice were intravenously administered PGSP or PGSP@Res, and renal histology, oxidative injury and biochemical indices were comprehensively analyzed.

**Figure 3:**
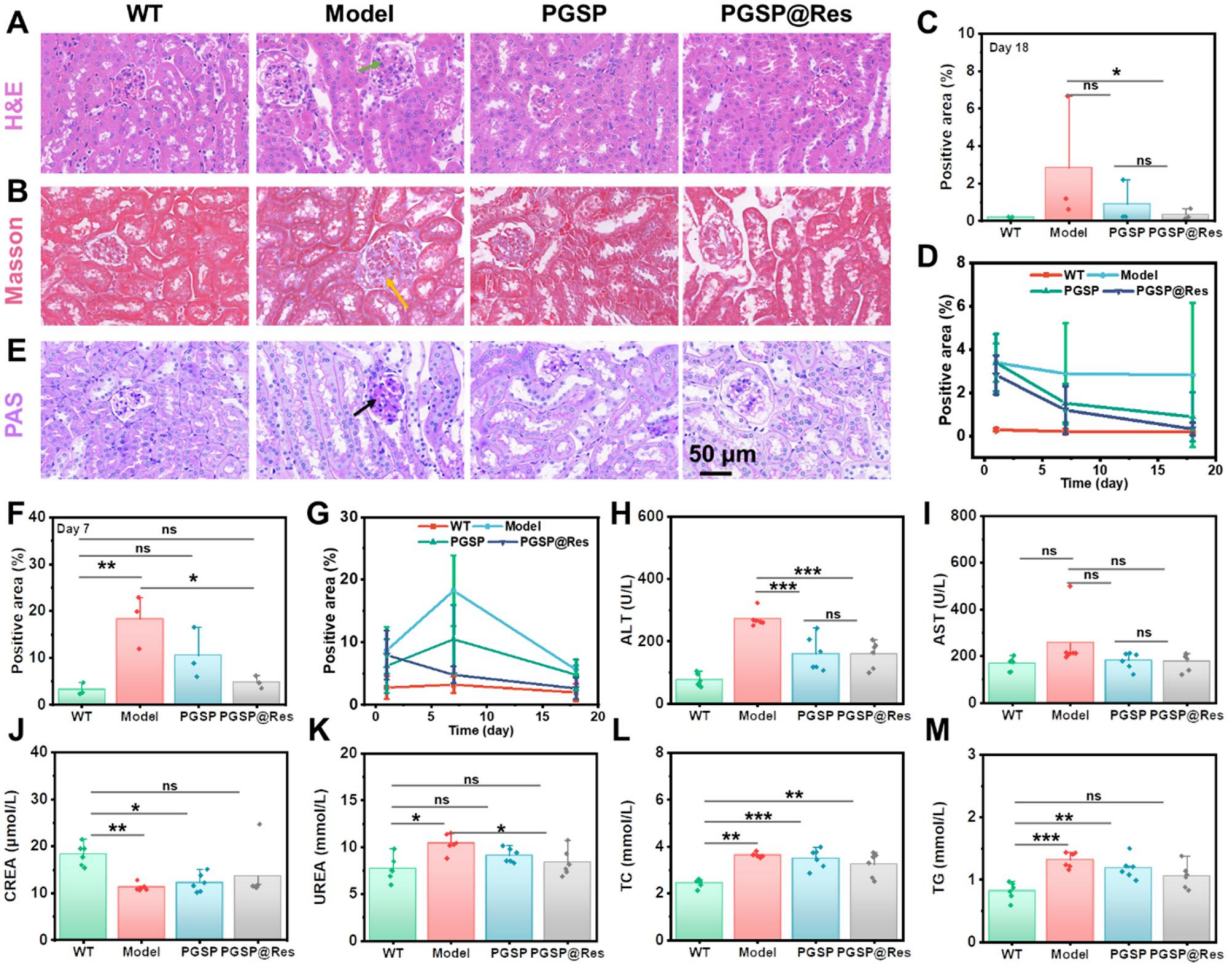
*In vivo* therapeutic efficacy of PGSP@Res NPs in DN. (**A**) H&E, (**B**) Masson staining images of renal tissues from WT, Model, PGSP and PGSP@Res groups at Day 18. (**C, D**) Quantitative analysis of fibrosis-positive area in Masson-stained sections at Day 18 and during the treatment period (*n* = 3). (**E**) PAS staining images of renal tissues from WT, Model, PGSP and PGSP@Res groups at Day 18. (**F, G**) Quantitative analysis of PAS-positive area at Day 7 and over time (*n* = 3). (**H**) ALT, (**I**) AST, (**J**) CREA, (**K**) UREA, (**L**) TC and (**M**) TG levels in various groups (*n* = 6). The ns represents no significant difference; *P < 0.05; **P < 0.01; ***P < 0.001.

H&E staining images revealed typical pathological alterations in the Model group, including glomerular mesangial expansion and tubular epithelial degeneration, indicative of diabetic renal injury (Figure 3A). These lesions persisted throughout the 18 days observation period without treatment. In contrast, PGSP treatment partially alleviated glomerular hypertrophy and mesangial proliferation by Day 18, whereas PGSP@Res treatment achieved substantial recovery as early as Day 7, with renal histology comparable to the WT group (Supplementary Figure S11). These results highlighted the accelerated structural restoration induced by the synergistic antioxidative therapy. Masson’s trichrome staining images (Figure 3B, and Supplementary Figure S12) further confirmed that the Model group exhibited excessive collagen deposition, a hallmark of renal fibrosis. Quantitative analysis (Figure 3C and D and Supplementary Figure S13) demonstrated that PGSP@Res markedly reduced collagen accumulation, achieving near-normal levels by Day 18. This pronounced antifibrotic effect likely arisen from the action of polyphenolic radical scavenging and Res-mediated suppression of profibrotic signaling pathways, consistent with previous reports linking oxidative stress to renal fibrogenesis [16]. Periodic acid-Schiff (PAS) staining (Figure 3E and Supplementary Figure S14) was performed to assess glycogen deposition within renal tissues. The Model group exhibited significant glycogen accumulation, reaching its maximum at Day 7, reflecting abnormal glucose metabolism in DN. Remarkably, PGSP@Res treatment facilitated rapid clearance of glycogen deposits, restoring nearly normal PAS staining intensity by Day 7 (Figure 3F and G and Supplementary Figure S15). In contrast, spontaneous recovery in untreated Model mice required over 18 days, confirming that PGSP@Res could accelerate metabolic homeostasis and renal functional recovery.

Given the systemic metabolic dysfunction associated with DN, hepatic and renal biochemical indices were analyzed to assess functional improvement. ALT and AST levels were measured to evaluate hepatic status. As shown in Figure 3H and I, the Model group displayed elevated ALT, indicating hepatic stress likely secondary to chronic hyperglycemia. Both PGSP and PGSP@Res treatments significantly reduced ALT levels, with PGSP@Res achieving near-normal values, while AST changes were less pronounced but followed a similar downward trend. These results suggested that the therapy not only mitigated renal injury but also alleviated systemic hepatic burden. Renal function markers, including CREA and UREA, were also examined (Figure 3J and K). Interestingly, the decreased CREA levels observed in the Model group may be attributed to glomerular hyperfiltration in early DN, where elevated filtration activity can transiently reduce circulating CREA levels before progressive renal impairment occurs. In contrast, UREA levels were significantly elevated, likely reflecting the initial decline in renal excretory capacity. Treatment with PGSP partially corrected these abnormalities, whereas PGSP@Res fully normalized both indicators, showing no statistical difference from the WT group. The recovery of CREA and UREA underscored the efficient renal protection and filtration restoration achieved by PGSP@Res NPs. Additionally, serum lipid profiles were evaluated to probe metabolic regulation. The total cholesterol (TC) levels were substantially increased in Model mice (Figure 3L), consistent with dyslipidemia associated with diabetic renal pathology. Although PGSP and PGSP@Res both reduced TC levels, the changes were moderate, suggesting that cholesterol regulation may require longer treatment duration or combined modulation. In contrast, triglyceride (TG) levels were effectively normalized after PGSP@Res therapy, indicating improved lipid metabolism (Figure 3M).

Overall, the combined histological and biochemical analyses fully demonstrated that PGSP@Res NPs could effectively reverse the pathological features of DN while restoring hepatic and renal function to near-normal levels. The superior efficacy of PGSP@Res compared to PGSP alone underscored the synergistic contribution of encapsulated Res and the polyphenolic NPs in modulating oxidative stress and inflammatory pathways. Of particular note was that PGSP@Res exhibited a faster histological recovery rate in the early stages of treatment than PGSP alone, highlighting the synergistic effect of Res loading. Res has been widely reported to exert anti-inflammatory and anti-fibrotic effects by activating pathways [50–52], and its antioxidative capacity, combined with that of PGSP, worked synergistically on multiple pathological aspects of DN. Furthermore, the improvement in ALT levels in the treatment group suggests that PGSP@Res may also have the potential to alleviate diabetes-related liver damage, demonstrating its systemic beneficial effects. However, the relatively limited effect on TC regulation also indicates that for the complex metabolic disorders accompanying DN, a longer treatment period or combination therapy with other drugs targeting metabolism may be necessary to achieve optimal results.

### Regulation of oxidative stress, inflammation and apoptosis by PGSP@Res NPs

Given the established link between excessive ROS and the progression of DN, modulation of oxidative balance represents a central therapeutic strategy [53, 54]. To elucidate the underlying mechanisms of PGSP@Res NPs mediated renal protection, oxidative stress regulation, inflammatory cytokine expression and apoptosis-related signaling were systematically investigated during the treatment period.

**Figure 4:**
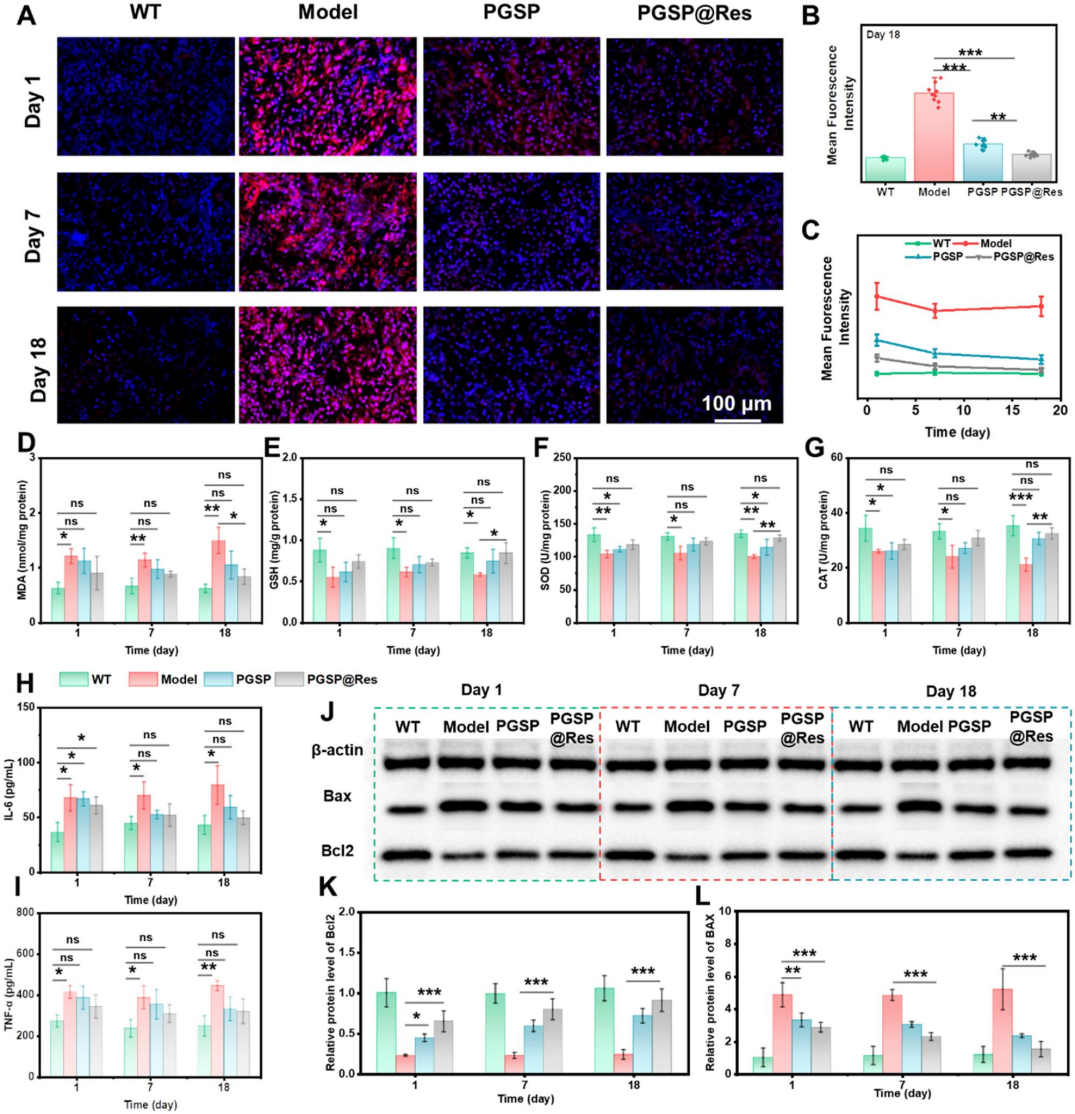
Regulation of oxidative stress, inflammation and apoptosis by PGSP@Res NPs during DN treatment. (**A**) The merged DHE staining images of renal tissues in various groups at different time points. (**B, C**) Quantitative and time-dependent analysis of DHE fluorescence intensity to show ROS levels with different treatments (*n* = 9). Levels of (**D**) lipid peroxidation marker MDA and (**E**) antioxidative molecule GSH, as well as endogenous antioxidant enzymes (**F**) SOD and (**G**) CAT (*n* = 3). Concentrations of proinflammatory cytokines (**H**) IL-6 and (**I**) TNF-α in renal tissue lysates (*n* = 3). (**J**) WB analysis of apoptosis-related proteins (Bcl-2 and Bax) at different time points. Quantification of (**K**) Bcl-2 and (**L**) Bax expression levels. The ns represents no significant difference; *P < 0.05; **P < 0.01; ***P < 0.001.

Dihydroethidium (DHE) staining was employed to directly visualize intracellular ROS accumulation within renal tissues. As shown in Figure 4A and Supplementary Figure S16–18, the Model group displayed sustained ROS overproduction throughout the experimental period, consistent with chronic hyperglycemia-induced oxidative stress. In sharp contrast, both PGSP and PGSP@Res markedly reduced ROS fluorescence intensity in a time-dependent manner, with PGSP@Res exhibiting the most pronounced suppression by Day 18. Quantitative fluorescence analyses (Figure 4B and C and Supplementary Figure S19) confirmed that PGSP@Res restored ROS levels close to those of the WT group, highlighting its superior oxidative regulatory capability. To further validate the redox modulation, MDA and GSH levels were quantified as representative markers of lipid peroxidation and intracellular antioxidative capacity, respectively. As shown in Figure 4D and E, the Model group exhibited elevated MDA and reduced GSH, reflecting oxidative damage and compromised antioxidative defense. Treatment with PGSP@Res NPs significantly decreased MDA content while restoring GSH to near-normal levels, indicating efficient inhibition of lipid peroxidation and reinforcement of buffering capacity. Enzymatic antioxidants were also assessed to evaluate endogenous defense activation. SOD activity showed no significant differences among groups at early time points, but increased notably after 18 days in the PGSP@Res group, suggesting unstained activation of the intrinsic antioxidative system (Figure 4F). CAT activity followed a similar trend, with gradual upregulation during treatment (Figure 4G). The enhanced SOD and CAT activities demonstrated that PGSP@Res not only scavenges ROS directly but also stimulates the intrinsic antioxidative pathway.

Since oxidative stress and inflammation are tightly intertwined in DN pathology, the levels of proinflammatory cytokines were next assessed. The expressions of IL-6 and TNF-α were markedly elevated in the Model group, confirming the establishment of an inflammatory microenvironment (Figure 4H and I). Both PGSP and PGSP@Res significantly downregulated these cytokines, with PGSP@Res showing stronger suppression at all time points, particularly by Day 18 when IL-6 and TNF-α levels nearly returned to baseline. This coordinated decline in inflammatory mediators underscored that equilibration effectively interrupted the feedback loop between oxidative stress and inflammation, mitigating renal inflammation. Oxidative stress driven apoptosis also contributes critically to renal structural degeneration in DN [55, 56]. To assess apoptotic regulation, the expression of apoptosis-related proteins Bcl-2 and Bax was examined by Western blotting (WB). As shown in Figure 4J, the Model group exhibited suppressed antiapoptotic Bcl-2 and elevated proapoptotic Bax, consistent with active apoptotic signaling. PGSP treatment partially restored the balance, while PGSP@Res treatment markedly enhanced Bcl-2 and suppressed Bax across all time points (Figure 4K and L). This regulation was attributed to the combined antioxidative and anti-inflammatory effects of PGSP@Res, which collectively reduce ROS-triggered mitochondrial dysfunction and downstream caspase activation.

### Restoration of renal function and structural integrity by PGSP@Res treatment

Given the demonstrated antioxidative, anti-inflammatory and antiapoptotic effects of PGSP@Res, the restoration of renal function and structural integrity were next sought in DN. A comprehensive assessment combining biochemical indicators and protein-level analyses was conducted to reveal the therapeutic mechanisms underlying renal protection.

**Figure 5:**
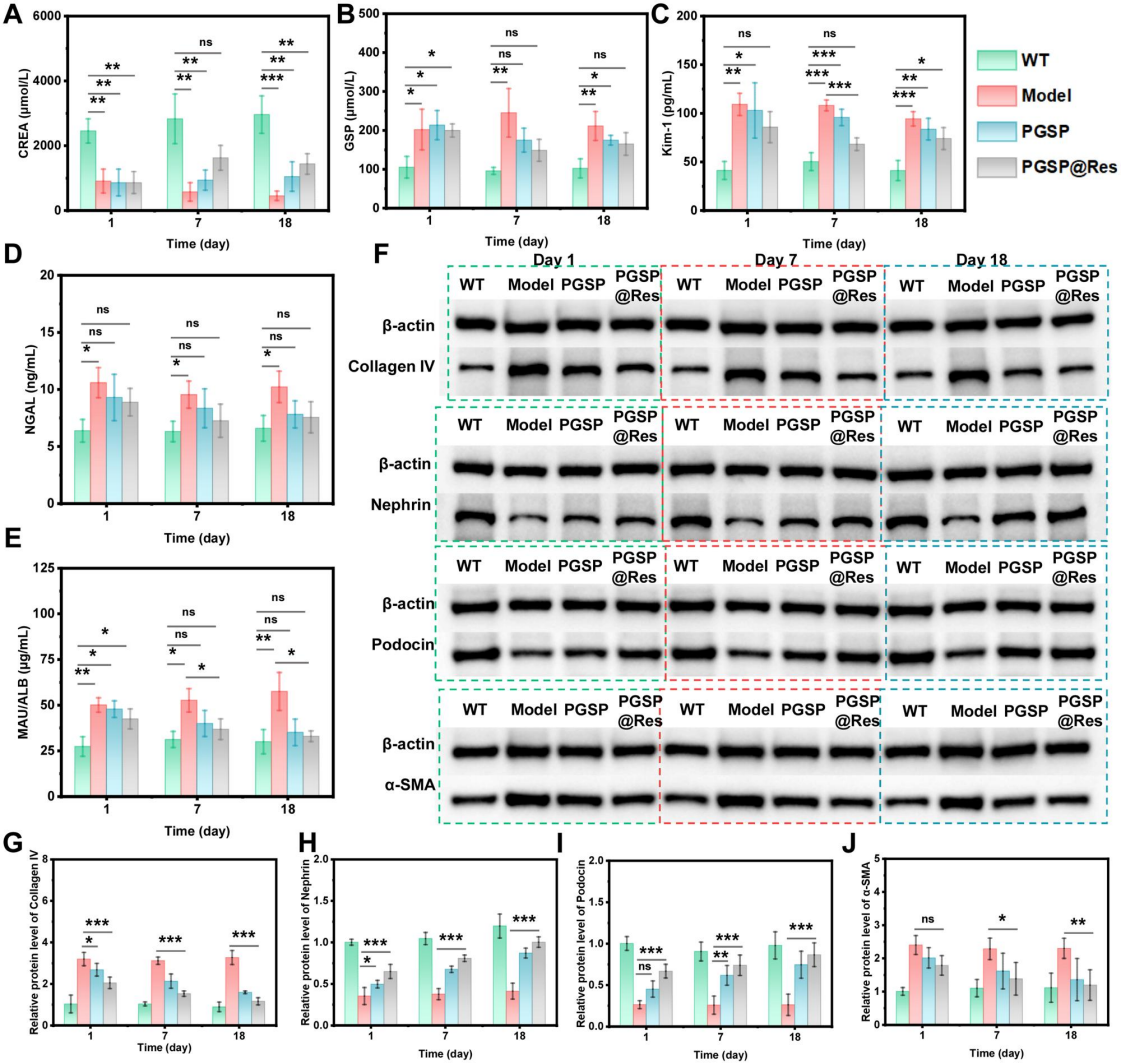
Restoration of renal functional and structural biomarkers in DN with PGSP@Res treatment. (**A**) CREA, (**B**) GSP, (**C**) Kim-1, (**D**) NGAL and (**E**) MAU/ALB levels in various groups at different time points (*n* = 3). (**F**) Representative WB analyses of Collagen IV, Nephrin, Podocin and α-SMA proteins in renal tissues at different time points. Quantified protein expression levels including (**G**) Collagen IV, (**H**) Nephrin, (**I**) Podocin and (**J**) α-SMA. The ns represents no significant difference; *P < 0.05; **P < 0.01; ***P < 0.001.

As shown in Figure 5A, CREA, a principal index of glomerular filtration capacity, was significantly decreased in DN model mice, which may be attributed to glomerular hyperfiltration in the early stage of DN rather than impaired filtration. This consistency also corroborated the rationality of the observed CREA changes. Following administration of PGSP or PGSP@Res, CREA levels progressively increased in a time-dependent manner, reflecting gradual functional restoration of the glomeruli. Consistently, GSP, a mid-term glycemic biomarker associated with hyperglycemia-induced oxidative damage, followed the same trend (Figure 5B). The reduction of GSP after PGSP@Res treatment indicated that the NPs not only alleviated oxidative injury but also indirectly contributed to improved metabolic regulation and glycemic stability. We further examined Kim-1, a sensitive biomarker for proximal tubular injury. The Model group showed elevated Kim-1 levels, confirming tubular epithelial stress and renal inflammation. PGSP@Res treatment rapidly restored Kim-1 to normal values as early as Day 1 (Figure 5C), demonstrating that the regulated microenvironment created by the polyphenolic nanoplatform enabled early structural repair and prevention of further injury. Additional renal injury markers, including NGAL and the MAU/ALB, exhibited similar responses (Figure 5D and E). Both indicators, elevated in the untreated Model group, decreased markedly following PGSP@Res administration, approaching levels comparable to the WT group at Day 18. These results collectively confirmed that PGSP@Res NPs effectively preserved tubular integrity and reduces glomerular leakage, thereby reestablishing renal filtration homeostasis.

To further elucidate the structural basis of functional recovery, the expression of representative renal and fibrotic proteins was analyzed by WB assays (Figure 5F). Collagen IV, a major extracellular matrix component associated with renal fibrosis, was markedly overexpressed in the Model group but was significantly reduced after PGSP@Res treatment (Figure 5G), suggesting inhibition of fibrogenic remodeling. In contrast, the podocyte-associated proteins Nephrin and Podocin, both of which are critical for maintaining the slit diaphragm and glomerular barrier [8, 57], were substantially downregulated in the Model mice but progressively restored in PGSP@Res-treated kidneys (Figure 5H and I). This recovery indicated that the nanoplatform preserved podocyte viability and stabilized cytoskeletal architecture through oxidative stress suppression and inflammatory modulation. Moreover, α-SMA, a hallmark of myofibroblast activation and fibrosis progression, was significantly upregulated in diabetic kidneys but dramatically downregulated after PGSP@Res treatment (Figure 5J). The concurrent normalization of Collagen IV and α-SMA expression highlighted the pronounced antifibrotic capacity of the nanocomposite, consistent with the histological restoration observed in Figure 3B. Together, these findings demonstrated that PGSP@Res treatment achieves a coordinated improvement of renal biomarkers, structural proteins and histological morphology. The time-dependent regulation of CREA, GSP, Kim-1, NGAL and MAU/ALB reflected functional recovery of both the glomerular filtration barrier and tubular reabsorption capacity, while protein-level analyses confirmed suppression of fibrosis and repair of podocyte integrity. This integrated recovery highlighted PGSP@Res NPs as a potent therapeutic system capable of addressing the multifactorial pathology of DN.

Functional recovery inevitably relied on structural repair. Our analysis of a series of key renal proteins explained the improvement in renal function indicators at the molecular level. The decrease in Collagen IV and α-SMA expression directly demonstrated that PGSP@Res NPs possessed a significant anti-fibrotic effect, delaying the key pathological process of DN progressing to end-stage renal disease. The restoration of expression of podocyte key proteins Nephrin and Podocin was of paramount importance. Podocyte injury and shedding are the core causes of proteinuria in the early stages of DN. PGSP@Res can repair structural proteins in podocyte slit diaphragms, indicating that it can directly protect the integrity of the glomerular filtration barrier, providing the most direct explanation for the improvement in urinary protein indicators. Simultaneously, the rapid return to normal levels of renal tubular injury markers Kim-1 and NGAL indicated that this treatment also possessed a strong protective effect on the renal tubules. In summary, PGSP@Res, through its powerful antioxidative capabilities, achieved dual protection for both glomeruli and renal tubules and effectively inhibited the fibrosis process, thereby reversing the pathological changes of DN in multiple dimensions.

## Conclusion

In this work, an enzymatically polymerized polyphenolic nanoplatform PGSP@Res was developed that combined intrinsic antioxidative activity with bioactive Res delivery for DN therapy. The HRP mediated polymerization strategy generated stable and biocompatible polyphenolic NPs, overcoming the solubility and instability limitations of natural polyphenols. Benefiting from synergistic radical scavenging and bioactive effects, PGSP@Res NPs efficiently alleviated oxidative stress, suppressed inflammation and reduced apoptosis. In DN model mice, PGSP@Res markedly improved renal morphology and function, restoring glomerular and podocyte structural proteins including CREA, Nephrin, Podocin and α-SMA for potential long-term benefits. This work provided a versatile, bioinspired nanotherapeutic platform for oxidative modulation and offered a promising strategy for treating DN and other oxidative stress related diseases.

## Supplementary Material

rbag102_Supplementary_Data

## Data Availability

The data can be obtained directly from the corresponding author.
