# Supplementary material for: Bioinspired enzymatic polyphenolic nanoplatform for redox modulation and functional renal restoration in diabetic nephropathy
Source: Regen Biomater. 2026 May 26;13:rbag102. doi: 10.1093/rb/rbag102 (PMC13303293; doi:10.1093/rb/rbag102)
Supplement: rbag102_Supplementary_Data [file rbag102_supplementary_data.docx]

**Bioinspired Enzymatic Polyphenolic Nanoplatform for Redox Modulation and Functional Renal Restoration in Diabetic Nephropathy**

Linli Cai^1,5^ , Tianyou Wang^2^, Yin Huang^3^, Dehong Cao^3^, Fenghao Yang^4^, Xingyuan Li^1,5^ , Yutong Zou^1,5^ , Qing Yang^1^, Maoyun Li^6^, Zhipeng Gu^2,^*, Fang Liu^1,5,^*

^1^Department of Nephrology, West China Hospital of Sichuan University, Chengdu 610041, China.

^2^College of Polymer Science and Engineering, State Key Laboratory of Advanced Polymer Materials, Sichuan University, Chengdu 610065, China.

^3^Department of Urology, West China Hospital of Sichuan University, Chengdu 610041, China.

^4^Department of Clinical Medicine, Southwest Medical University, Luzhou, 646000, China.

^5^Laboratory of Diabetic Kidney Disease, Kidney Research Institute, Department of Nephrology, West China Hospital of Sichuan University, China.

^6^Huaxi MR Research Center, Department of Radiology, Frontiers Science Center for Disease-Related Molecular Network, State Key Laboratory of Biotherapy, West China Hospital, Sichuan University, Chengdu, 610041, China.

E-mail: guzhipeng2019@scu.edu.cn (Z. G.); liufangfh@163.com (F. L.)

**Supplementary Experimental section**

*Characterization of PGSP and PGSP@Res NPs*

The morphology of the NPs was examined by SEM (Phenom Pro microscope). Samples were prepared by dropping dilute aqueous dispersions of nanoparticles onto freshly cleaved mica substrates, followed by gentle spinning to remove excess solvent and complete drying under ambient conditions. The hydrodynamic diameter and zeta potential were measured using a Malvern Zetasizer Nano ZS ZEN3690. Each sample was analyzed in triplicate, with the average values obtained from 12 consecutive runs in deionized water at 25 °C. UV-visible absorption spectra were recorded on a PerkinElmer Lambda 650 spectrophotometer with a 2 nm slit width. FTIR was performed using a Perkin-Elmer Spectrum One B system with the KBr pellet method at a resolution of 4 cm^-1^. XPS analyses were carried out on a PHI Quantera SXM spectrometer using Al Kα radiation. The pass energy was set to 160 eV for survey scans and 20 eV for high-resolution O 1s spectra with a dwell time of 300 ms.

*Evaluation of Free Radical Scavenging Activity*

The antioxidative capacity of PGSP and PGSP@Res NPs was evaluated *via* DPPH and ABTS radical scavenging assays to assess reactivity in both organic and aqueous environments. For DPPH assay, 1.0 mM ethanolic DPPH solution was freshly prepared prior to each test. NPs dispersions were prepared at 1 mg/mL. During the assay, 100 μL of NPs dispersion and 300 μL of DPPH solution were added into 2.6 mL of ethanol to form a homogeneous mixture exhibiting an initial purple color. The decrease in absorbance at 517 nm was monitored over time using a UV-Vis spectrophotometer to evaluate radical scavenging efficiency and reaction kinetics. All measurements were conducted in triplicate, and the scavenging percentage was calculated.

For ABTS assay, a 7 mM aqueous ABTS solution was oxidized with 2.45 mM potassium persulfate to generate ABTS^+·^ radicals, followed by incubation in the dark for 12 h. A 1 mg mL⁻¹ NPs solution was then prepared in deionized water. For the test, 100 μL of ABTS^+·^ solution and 100 μL of NPs dispersion were mixed with 2.8 mL of deionized water. The absorbance decrease at 714 nm was recorded at different time intervals to determine the scavenging efficiency. All experiments were repeated three times, and the average results were used for analysis.

*In vitro release of Res*

The *in vitro* release behavior of resveratrol from PGSP@Res NPs was evaluated using a dialysis method. Briefly, 1.0 mL of PGSP@Res NPs dispersion (3 mg/mL) was transferred into a dialysis bag (molecular weight cut-off, MWCO: 3.5 kDa), which was then immersed in 60 mL of phosphate-buffered saline (PBS, pH 7.4) in a sealed amber bottle to simulate physiological conditions and avoid light-induced degradation. The system was maintained at 37 °C with continuous shaking at 100 rpm. At predetermined time intervals, 0.5 mL of the release medium was collected for analysis and replaced with an equal volume of fresh PBS to maintain sink conditions. The amount of released resveratrol was quantified by measuring the absorbance at 306 nm using a UV-vis spectrophotometer, and the cumulative release profile was calculated accordingly.

*Cell Viability Assay*

HK-2 cells were digested, counted, and resuspended in complete culture medium to form a single-cell suspension. Cells were seeded into 96-well plates at an appropriate density and preincubated for 24 h at 37 °C and 5 % CO_2_ to allow adherence and entry into the logarithmic growth phase. For cytotoxicity evaluation, cells were treated with different concentrations of PGSP or PGSP@Res for 48 h. For therapeutic evaluation under oxidative stress, cells were co-treated with materials and 75 μM H_2_O_2_ for 24 h. After the designated treatments, 10 μL of Cell Counting Kit-8 (CCK-8, Biosharp) solution was added directly to each well under light-protected conditions. The plate was gently agitated to ensure uniform mixing and incubated for 1-4 h. The absorbance at 450 nm was measured using a microplate reader (Multiskan FC, Thermo Fisher Scientific). Cell viability was calculated relative to untreated controls.

*Intracellular ROS Detection*

Intracellular ROS levels were detected using a DHE fluorescence probe. After treatment, cells grown on coverslips were washed 1-3 times with PBS (pH 7.4), then incubated with a freshly prepared DHE solution at 37 °C for 20 min in the dark. After staining, cells were washed three times with PBS on a shaker for 5 min each and mounted with antifade reagent. Fluorescence was observed using an excitation wavelength of 518 nm and an emission wavelength of 610 nm under a fluorescence microscope (Nikon Eclipse C1).

*Acute Toxicity Evaluation*

To assess the acute toxicity and systemic effects of the NPs, 8-week-old male C57BL/6J mice were acclimated for one week before treatment. Mice were randomly divided into several groups and administered *via* tail vein injection with PGSP or PGSP@Res at doses of 1, 2, 5, and 10 mg/kg, respectively; an equivalent volume of saline was used as a control. The injection volume was standardized at 5 mL/kg body weight. Mice were monitored continuously for 24 h after administration for general activity, feeding, and behavioral changes. At 24 h post-injection, animals were sacrificed, and blood, heart, liver, spleen, lung, and kidney tissues were collected for histological and biochemical analyses.

*Drug Administration and Sample Collection Procedures*

Mice were restrained in a dedicated fixation device, and the tail was sterilized and warmed with 75 % ethanol. The fuller of the two lateral tail veins was selected, and drug administration was performed using a 1 mL sterile insulin syringe (29G needle) inserted at an angle <30°. After confirming blood return, the drug solution was injected slowly, and the puncture site was gently compressed with a cotton ball to prevent bleeding. All collection procedures were performed after deep anesthesia with intraperitoneal sodium pentobarbital (1 %, 45 mg/kg). Euthanasia was achieved by cervical dislocation following sample collection. Blood was obtained *via* retro-orbital puncture and allowed to clot at room temperature for 30 min before centrifugation at 3000×g for 15 min at 4 °C to isolate serum, which was stored at -80 °C. Urine samples were collected immediately after urination under observation and stored at -80 °C. Major organs (heart, liver, spleen, lung, kidney, pancreas) were excised, washed with pre-cooled saline, blotted dry, and divided into portions: tissues for histopathology were fixed in 4 % paraformaldehyde, samples for molecular assays (ELISA, WB, qRT-PCR) were snap-frozen in liquid nitrogen and stored at -80 °C.


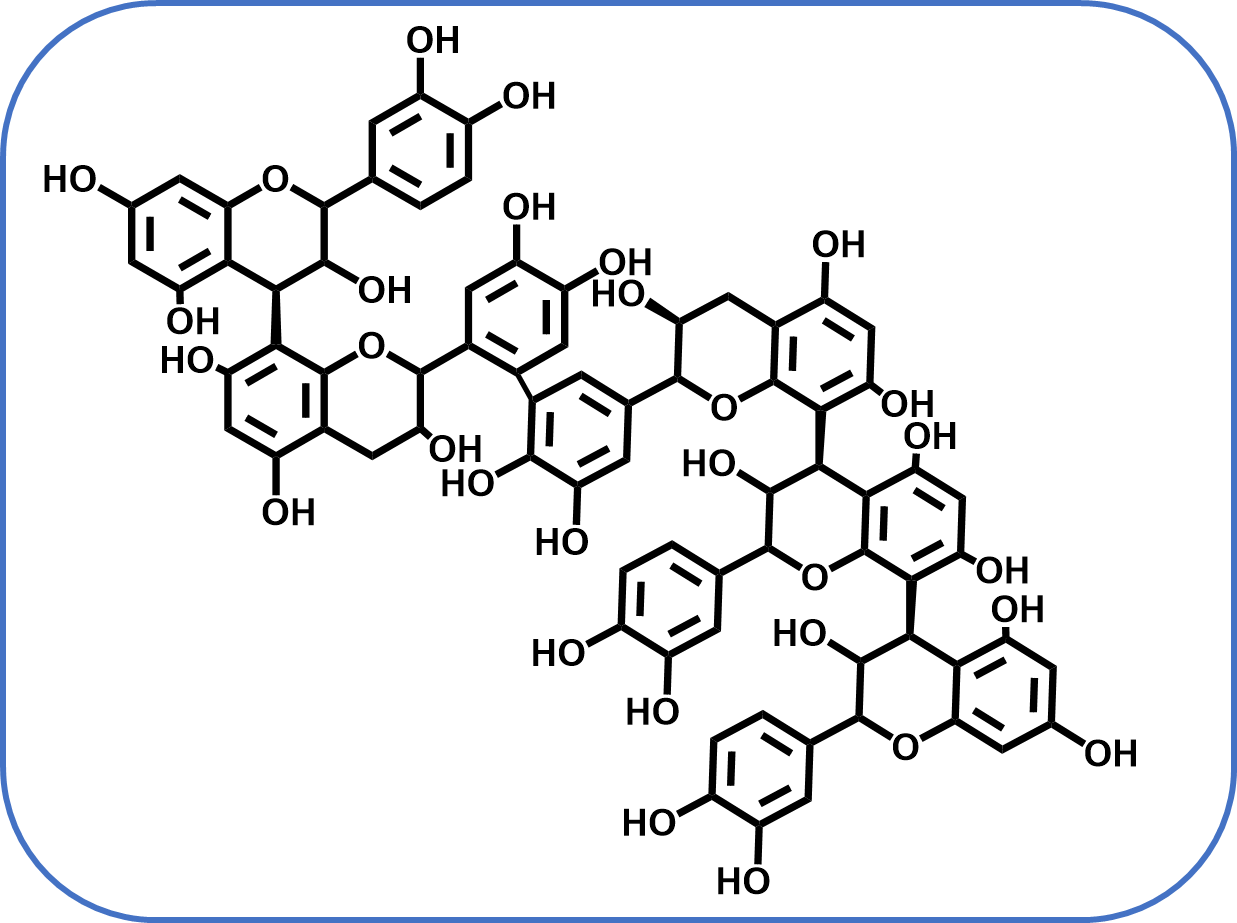


**Figure S1.** Structural formula of oligomeric units constituting PGSP NPs.


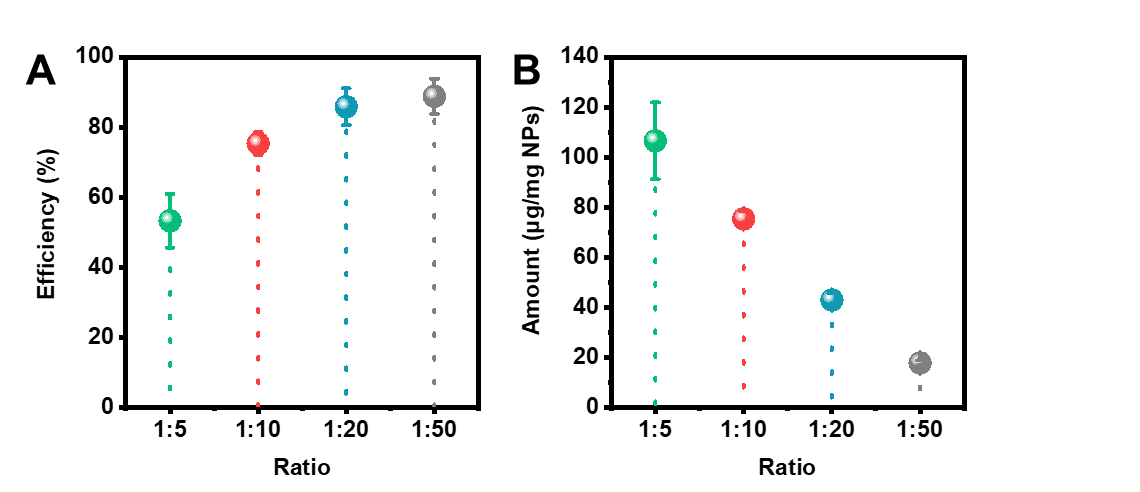


**Figure S2.** Optimization of Res (A) loading efficiency and (B) loading amount in PGSP@Res NPs.

**Figure S3.** The release profile of Res with time up to 72 h.


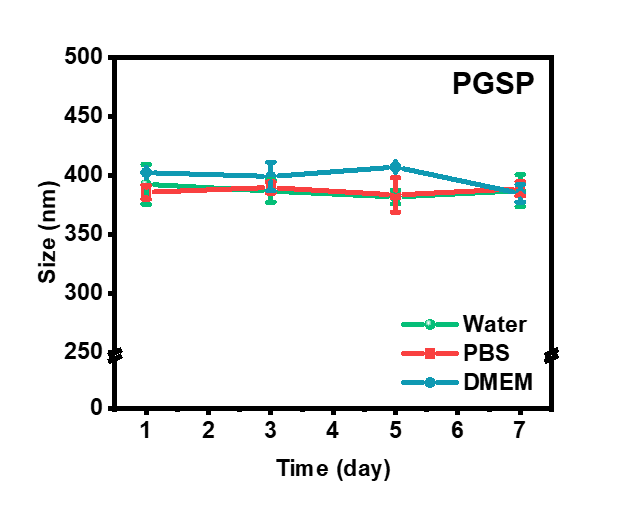


**Figure S4.** Colloidal stability of PGSP in different media (water, PBS and DMEM) over 7 days.


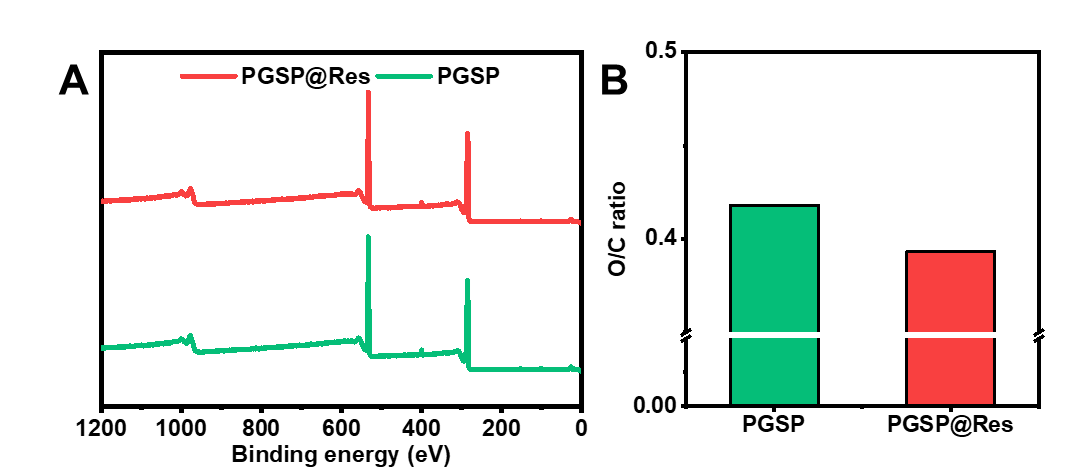


**Figure S5.** (A) XPS survey spectra and (B) O/C atomic ratio of PGSP and PGSP@Res.


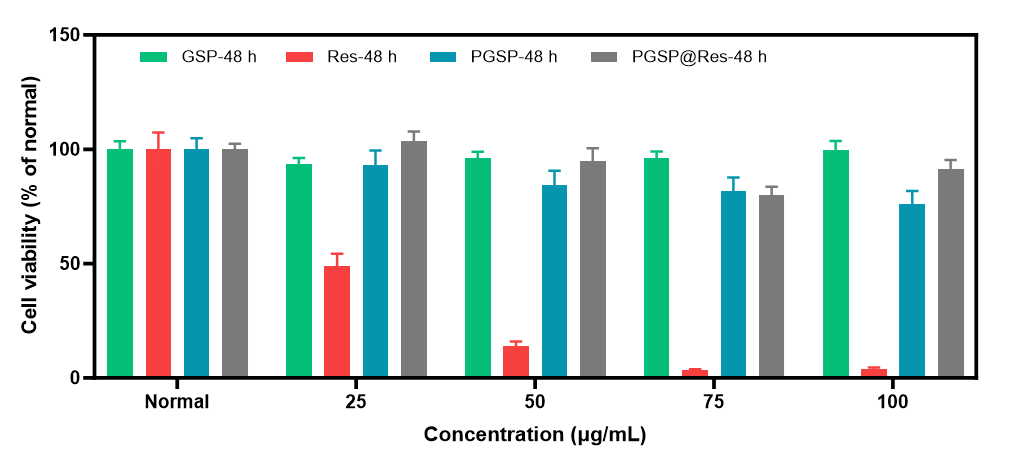


**Figure S6.** Cell viability of GSP, Res, PGSP, and PGSP@Res over a wide concentration range after 48 h.


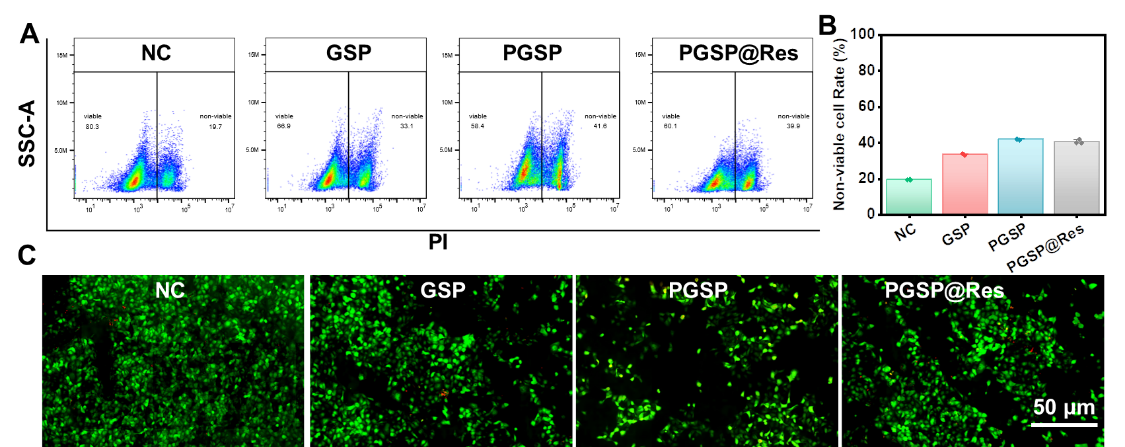


**Figure S7.** (A-B) Flow cytometry analysis and (C) live/dead staining images in various groups.


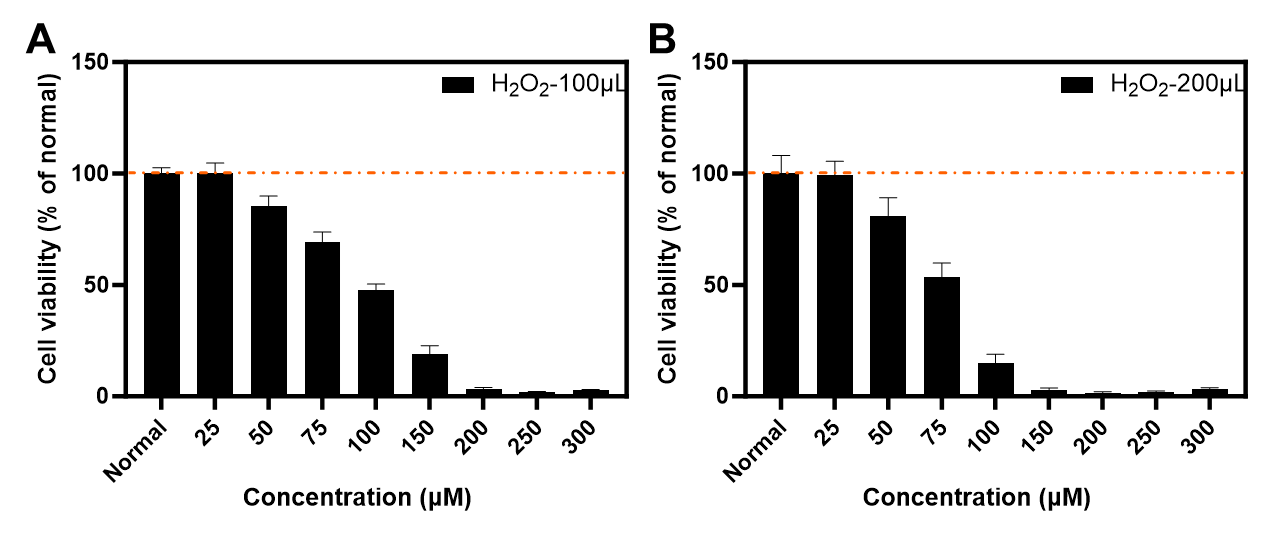


**Figure S8.** Optimization of H_2_O_2_ concentration and dosage for inducing oxidative stress *in vitro*.


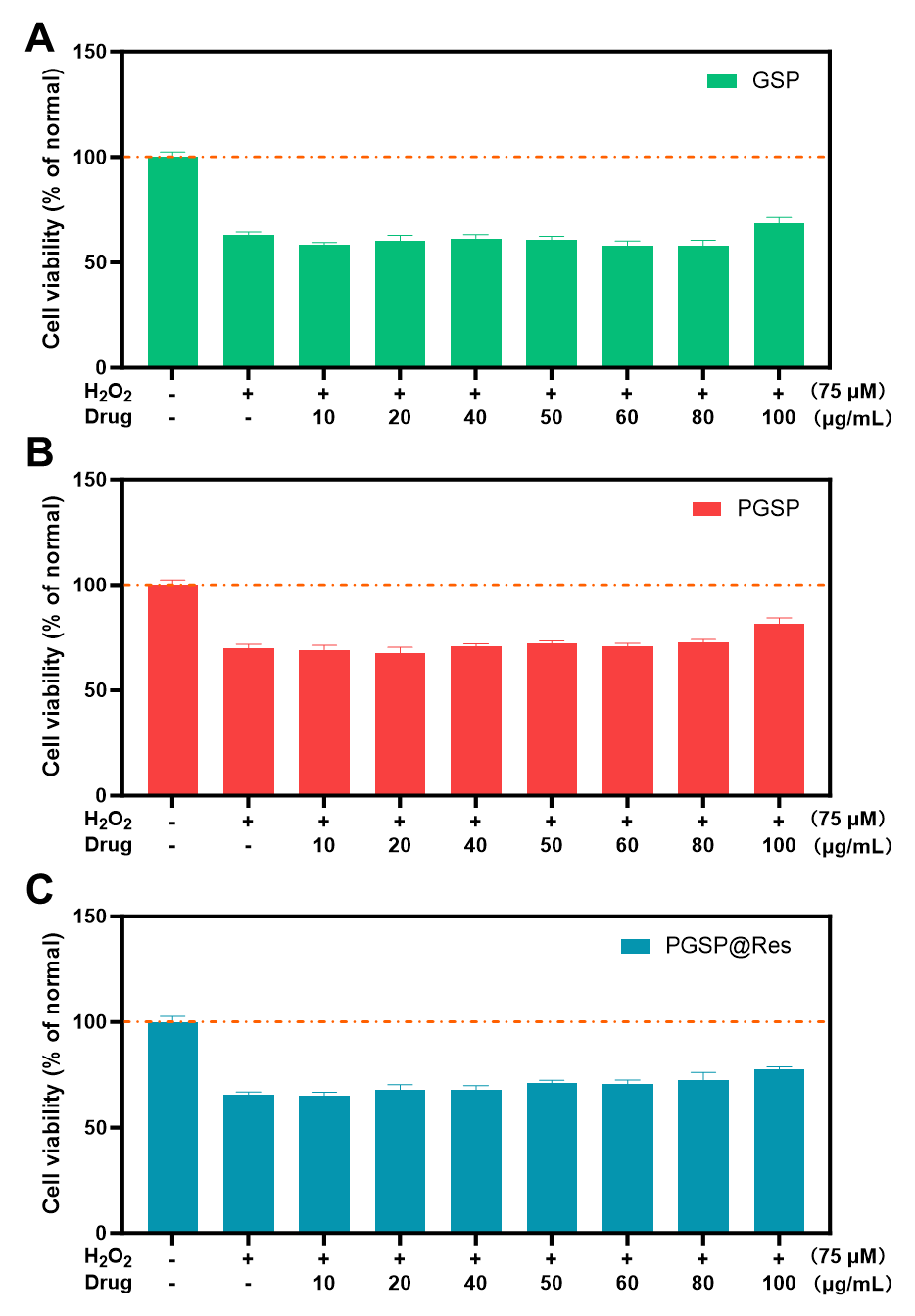


**Figure S9.** Quantitative cell viability assays after GSP, PGSP or PGSP@Res NPs treatment under oxidative stress.


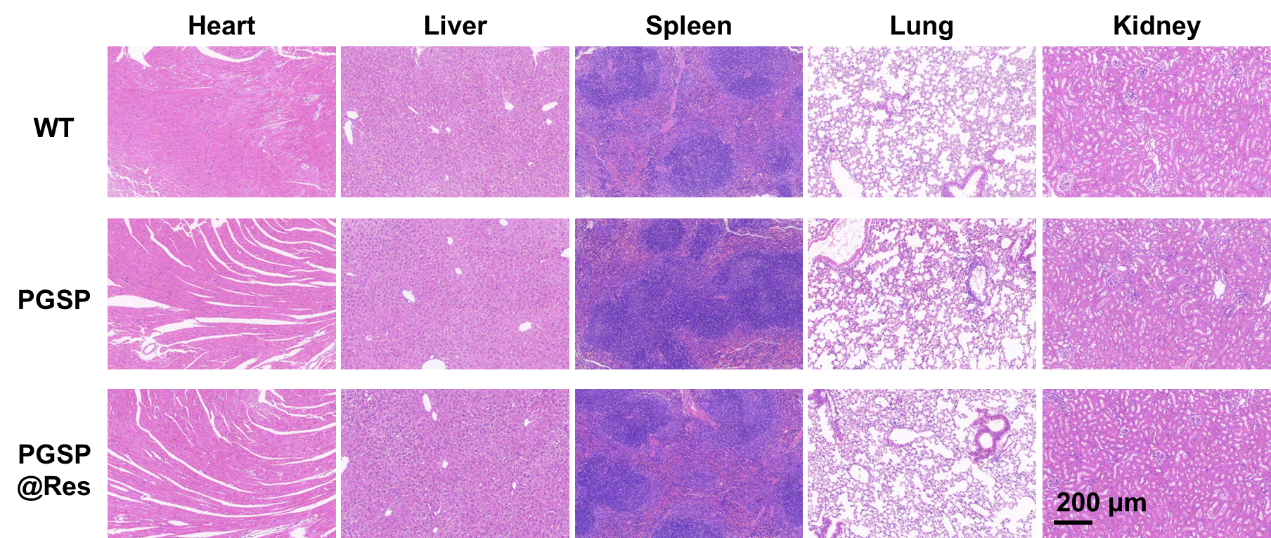


**Figure S10.** Histological evaluation by H&E staining images of heart, liver, spleen, lund and kidney confirming negligible tissue toxicity of PGSP and PGSP@Res.


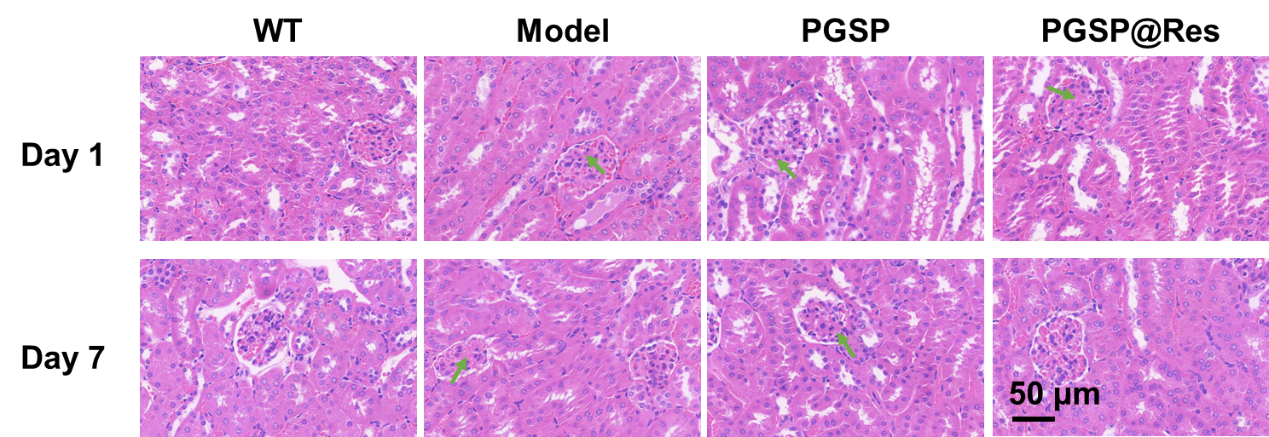


**Figure S11.** Additional H&E staining images of renal sections at multiple time points.


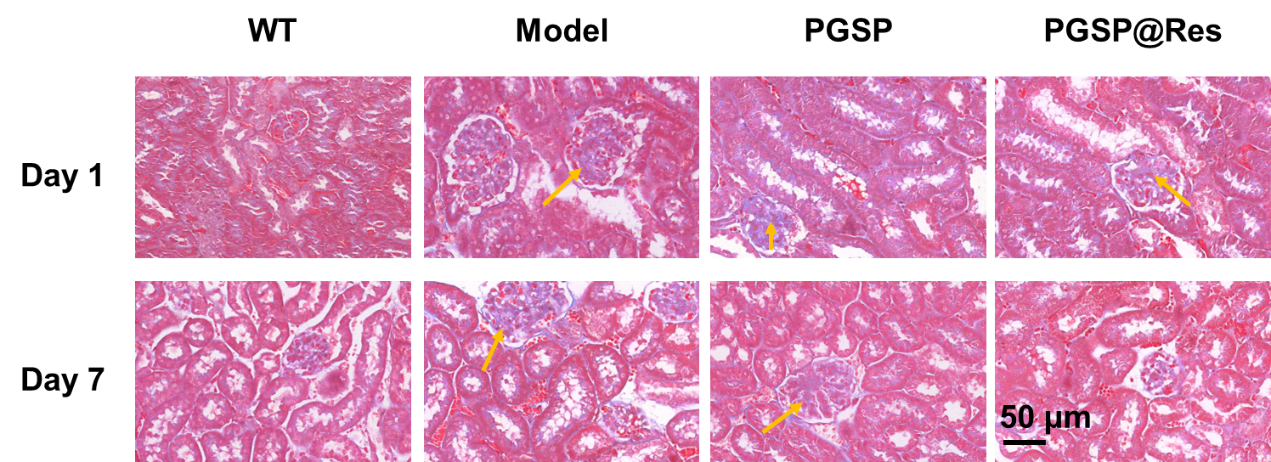


**Figure S12.** Additional Masson staining images of renal sections at multiple time points.


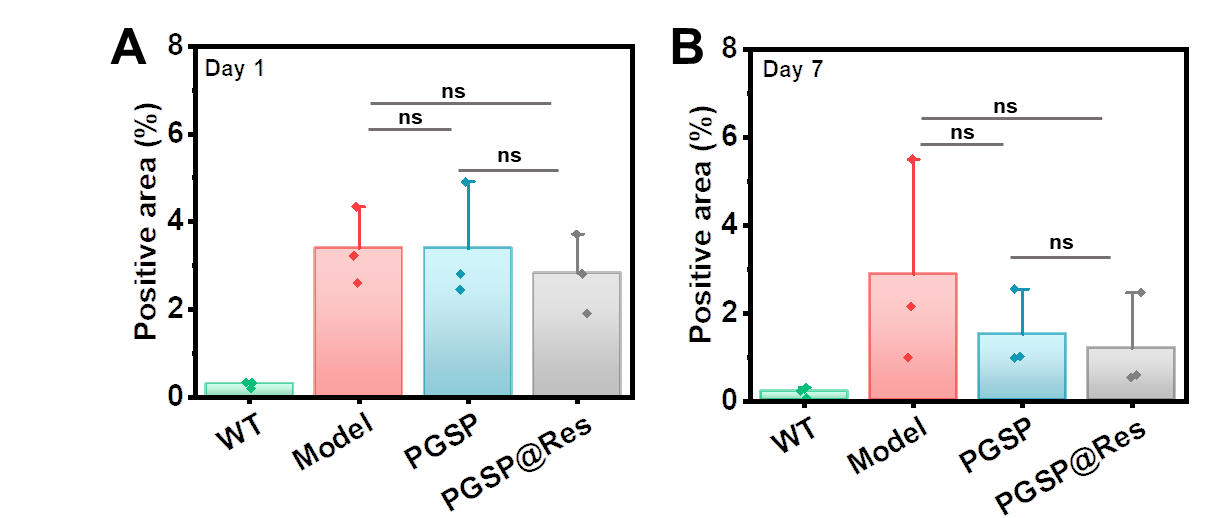


**Figures S13.** Extended quantitative analysis of collagen-positive area at multiple time points (n=3). The ns represents no significant difference; * represents p < 0.05; ** represents p < 0.01; *** represents p < 0.001.


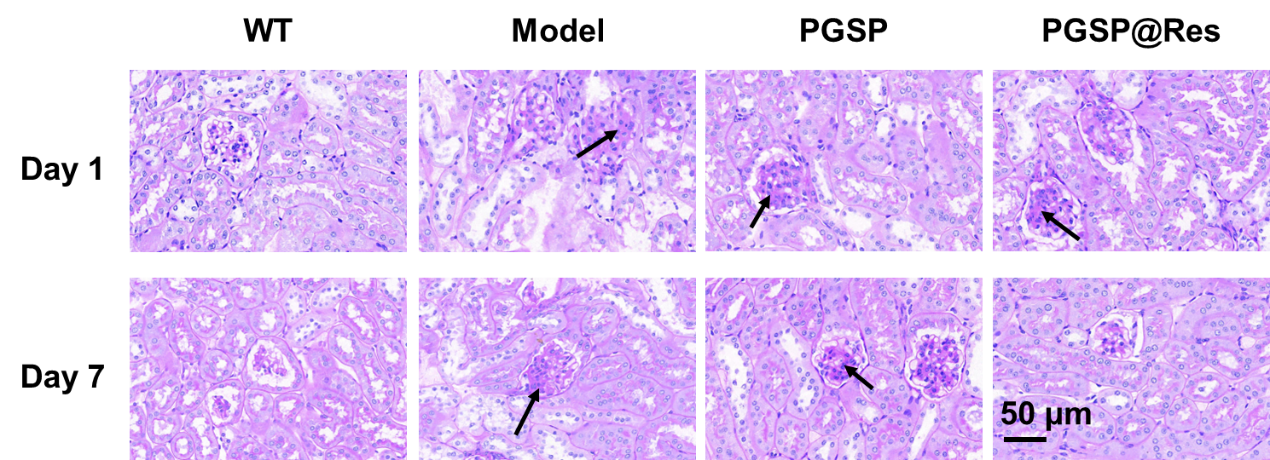


**Figure S14.** Additional PAS staining images of renal sections at multiple time points.


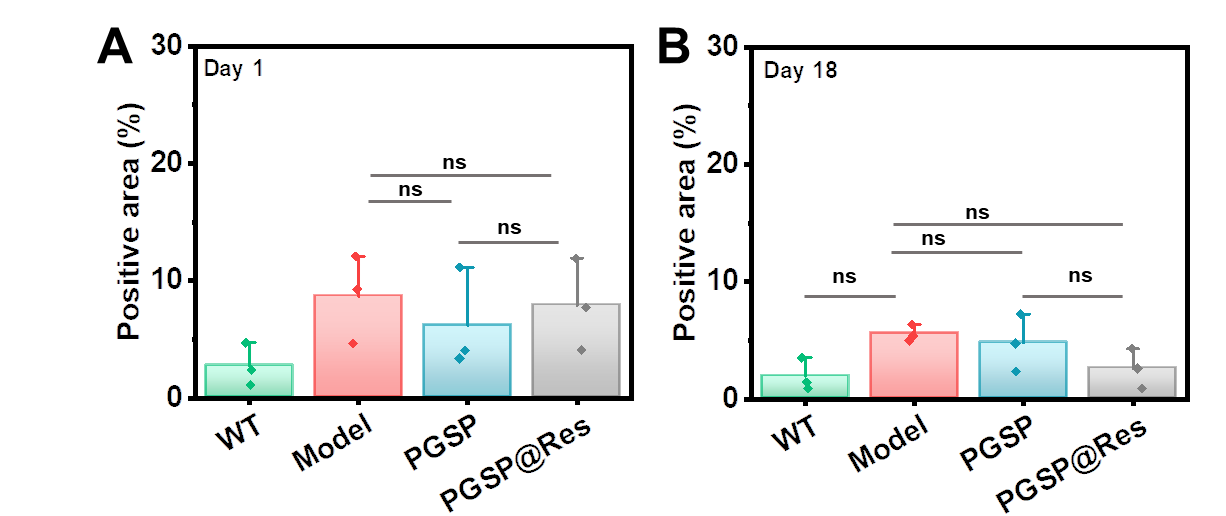


**Figures S15.** Extended quantitative analysis of glycogen-positive area at multiple time points (n=3). The ns represents no significant difference; * represents p < 0.05; ** represents p < 0.01; *** represents p < 0.001.


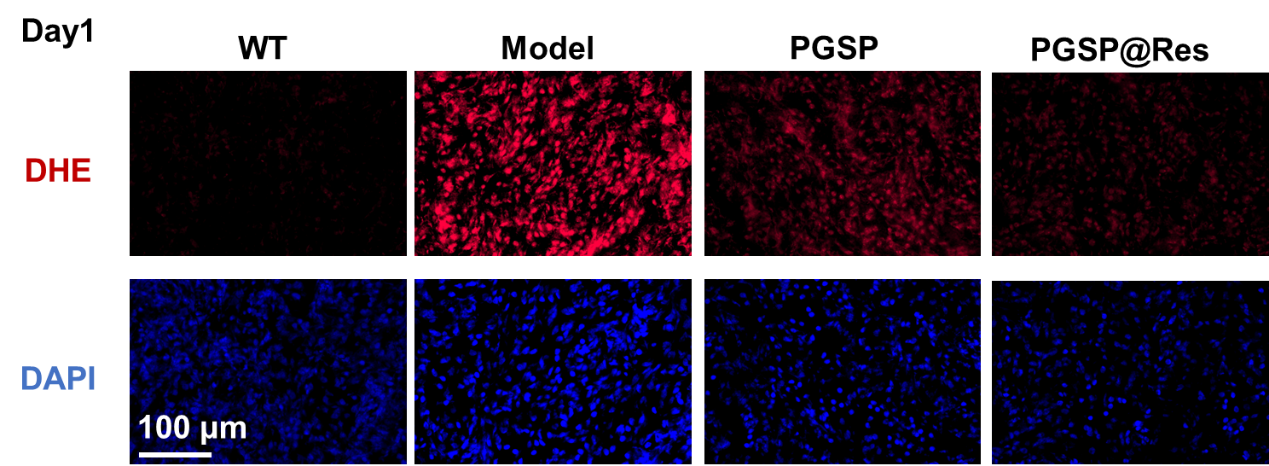


**Figure S16.** The DHE and DAPI staining images at day 1.


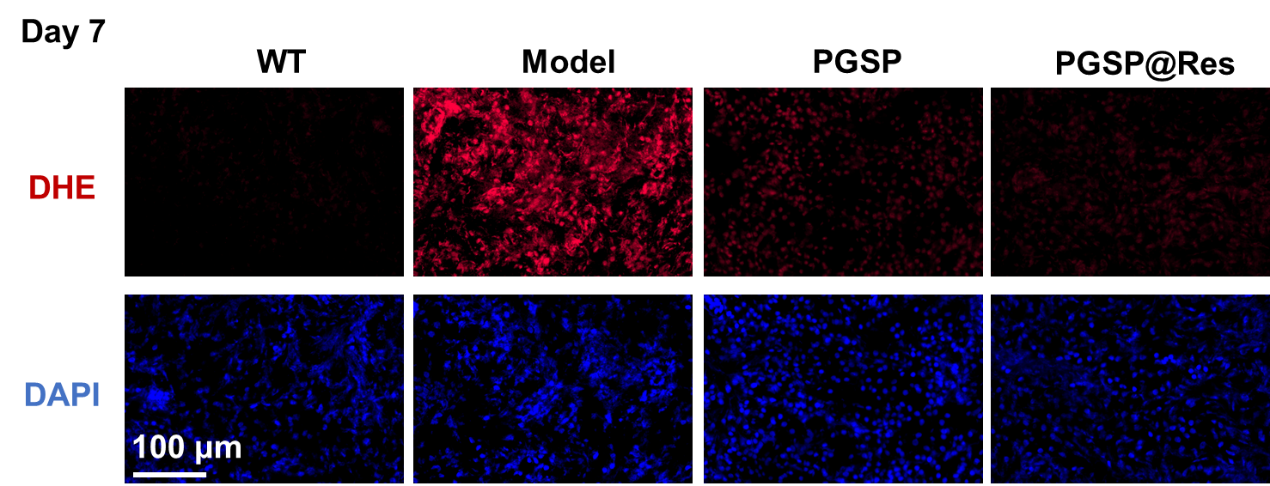


**Figure S17.** The DHE and DAPI staining images at day 7.


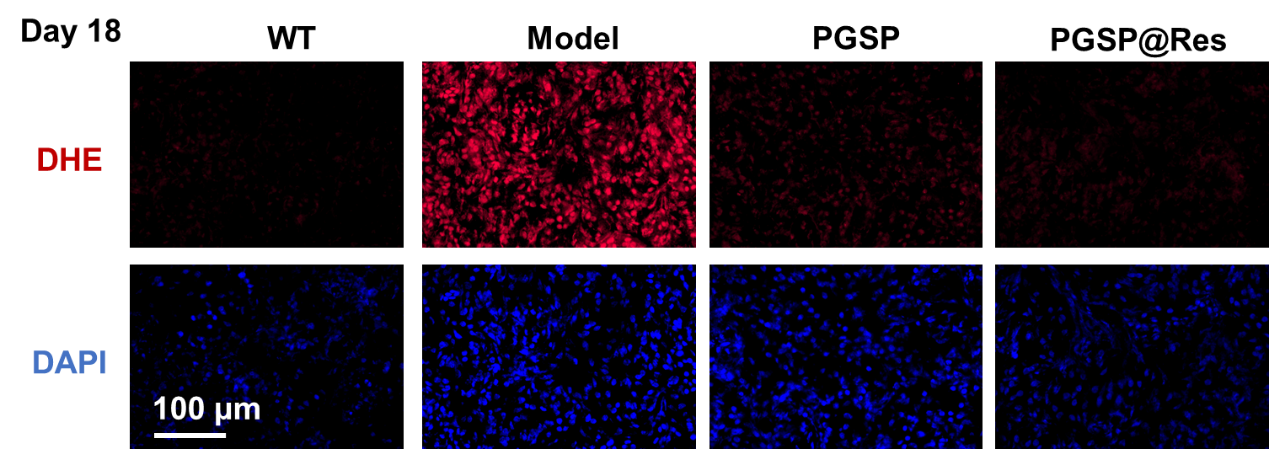


**Figure S18.** The DHE and DAPI staining images at day 18.


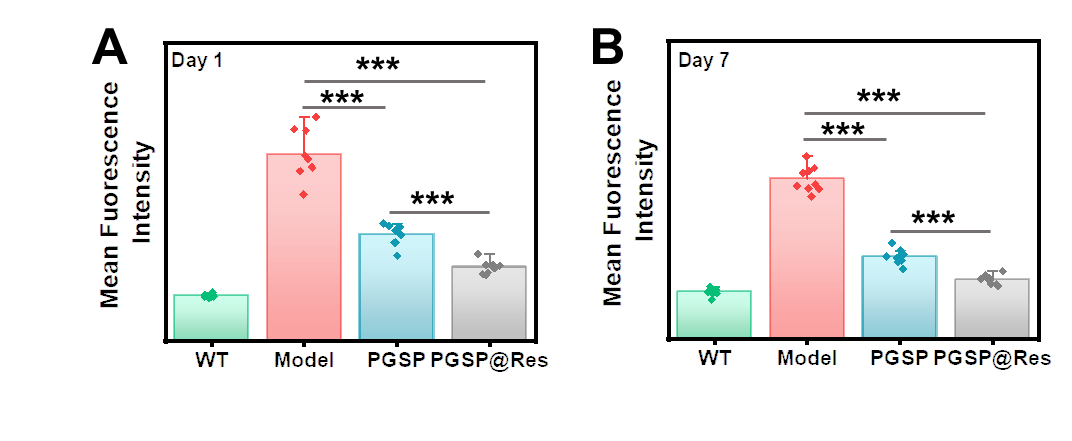


**Figure S19.** Extended DHE fluorescence quantification of ROS production at multiple time points (n=9). The ns represents no significant difference; * represents p < 0.05; ** represents p < 0.01; *** represents p < 0.001.

**Table S1.** Sequences of primers in this study.

|  | Forward (5’-3’) | Reverse (5’-3’) |
| --- | --- | --- |
| Hmox1 | ACTGCGTTCCTGCTCAACAT | GGGGGCAGAATCTTGCACT |
| SOD2 | TAGCTCTTCAGCCTGCACTG | TTCCAGCAACTCCCCTTTGG |
| CAT | CTCCGGAACAACAGCCTTCT | ATAGAATGCCCGCACCTGAG |
| IL-6 | CCTTCTCCACAAGCGCCTTC | GGAAGGCAGCAGGCAACA |
| GAPDH | TGCACCACCAACTGCTTAGC | GGCATGGACTGTGGTCATGAG |
